# Task complexity in exoskeleton setup and takedown: Procedural steps and usability problems as predictors of deployment performance

**DOI:** 10.1371/journal.pone.0348001

**Published:** 2026-04-28

**Authors:** Jessica Sanchez-Balandran, Alejandra Martinez Fernandez, Laura Tovar, Priyadarshini Pennathur, Arunkumar Pennathur

**Affiliations:** Industrial, Manufacturing and Systems Engineering Department, Physical, Information and Cognitive Human Factors Engineering (PIC-HFE) Research Laboratory, Industrial, University of Texas at El Paso, El Paso, Texas, United States of America; King Fahd University of Petroleum & Minerals, SAUDI ARABIA

## Abstract

Occupational exoskeletons show promise in reducing physical strain in industrial work, yet their industrial adoption remains limited. For exoskeletons to be used when performing tasks in industrial settings, they first need to be set up, then fitted and donned, and finally doffed, disassembled and stored for future use. Exoskeleton setup and takedown procedures can significantly impact industry deployment, adoption, and use, yet we have limited knowledge of the complexities in setup and takedown procedures and the resulting deployment barriers. The goals of this study were to understand how task complexity (number of task steps, usability problems and part count) in setup and takedown of exoskeletons impact task completion time and success, and present barriers in deployment. Twenty nine participants completed setup (assembly and donning) and takedown (doffing and disassembly) of four exoskeletons. We measured task times, success rates, task complexity (task step counts, part counts and usability problems). Hierarchical task analysis and heuristic assessments were performed to assess task complexity. Setup tasks, especially assembly, took the most time and exhibited the highest failure rates, whereas takedown tasks were faster and more successful. Participant-level regression analyses (N = 397 observations) showed that number of procedural steps was the strongest predictor, accounting for 66.9% of variance in completion times (β = 0.374, p < .001), with each additional step associated with approximately 22 seconds longer completion time. Usability problems also significantly predicted completion time (R² = 0.305, p < .001), while part count showed no significant association (p = .133). Our findings highlight that task complexity impact setup procedures significantly and can present a major deployment barrier. Moreover, our findings add new knowledge that number of steps, not part count, is the primary predictor of performance in setup procedures, and that usability problems differentially affect assembly and donning tasks.

## 1. Introduction and background

Wearable exoskeletons are emerging as a promising intervention to mitigate physical strain during demanding industrial and occupational tasks by augmenting human strength and reducing musculoskeletal loading [1–9]. Laboratory and field studies have demonstrated their potential to reduce spinal loading during lifting and lowering [10–17], and muscle activation during overhead work [6,9,18–22].

Despite these promising outcomes, real-world adoption of wearable exoskeletons remains limited due to practical challenges [3,4,7–9,23–31], underscoring the need to better understand the different barriers that currently inhibit their widespread adoption. Recent reviews highlight the barriers in usability, technological and regulatory constraints, socio-psychological factors, and economic factors. Usability challenges such as poor fit, restricted mobility, and discomfort during prolonged use, can undermine worker acceptance [7,24,32]. Technological limitations especially for powered exoskeletons include kinematic misalignment [32–34]. Furthermore, the absence of widely accepted regulatory standards and safety certification processes can make organizations hesitant to integrate exoskeletons into safety programs [35]. Worker resistance or skepticism [25,36,37] coupled with high upfront and maintenance costs, can further discourage exoskeleton adoption, particularly among small and medium-sized enterprises [37–39].

The barriers above studied in previous exoskeleton ergonomics research are typically encountered during procurement or operational use, but not during setup and takedown tasks that precede use. Setup and takedown tasks, including assembly, fitting, donning, doffing, disassembly, and storage, are essential for safe and reliable deployment but remain poorly understood. Customization of the device is often necessary to accommodate diverse users and work environments [40], necessitating careful assembly and calibration before use. Assembly involves integrating components such as sensors, actuators and control systems, particularly in active exoskeletons, where precise alignment is critical [41]. Calibration aligns the device to the user’s body and the specific task, adjusting control systems and contact pressures to maintain proper fit, comfort, and avoid misalignment [42,43]. Donning and doffing must also be intuitive, quick, and manageable by a single user; otherwise, the time burden and complexity can discourage consistent use [44]. Disassembly enables cleaning, maintenance and repairs to ensure hygiene, adaptability to different tasks or users, safety, longevity and protection from environmental hazards [45,46]. Moreover, regulatory and safety standards may require periodic setup and takedown to identify and mitigate hazards and ensure workplace safety [47]. Thus, setup and takedown are integral tasks in exoskeleton deployment that warrant systematic investigation to understand the factors influencing the complexity of these tasks. Understanding the complexity of these tasks requires examining not only their procedural steps, but also how usability and design challenges influence their execution.

The complexity of setup and takedown tasks often stems from usability problems that can increase physical effort, cognitive demand, and overall task time. Our usability studies have shown that setup and takedown tasks in exoskeletons are challenging and complex, with frequent usability problems such as unclear or incomplete instructions, the need for two-person operation, and non-intuitive device-user interaction points [48]. Donning and doffing tasks can carry safety hazards due to poor design, including risks of joint hyperextension, misalignment and rebound of heavy mechanical parts [48,49]. These tasks can also require considerable manual strength – for instance, activating controls sometimes requires both hands to engage mechanisms [48]. Achieving efficiencies in these tasks can be an additional barrier to overcome – lengthy or cumbersome donning and doffing processes may reduce acceptance, while designs that enable quick, independent use may be more likely to be adopted in occupational settings where productive time is critical [43,50].

When exoskeletons are viewed as a product, their product design architecture can also influence the complexities involved in their setup and takedown, and the resulting performance outcomes during these tasks. Research in the product design and design for assembly and disassembly literature demonstrates how product design architectures that increase structural and procedural complexities directly influences product assembly and disassembly performance. Poor part grouping within packaging can increase search time and frustration during assembly [51]. When designing a product, metrics such as part count, handling difficulties, fastening requirements, and topological interdependencies between parts have been linked to longer assembly and disassembly times [52–54]. Studies demonstrate a nonlinear increase in assembly and disassembly times and errors as product design complexity increases [55–57]. Design for Assembly guidelines, such as those codified by Boothroyd-Dewhurst method [58] further highlight how part symmetry, orientation during assembly and need for tools directly translate into assembly task complexities and inefficiencies. These frameworks suggest that product design complexities resulting from part count metrics and how the components in the product are structurally arranged and integrated, can directly impact its assembly and disassembly.

In summary, insights from literature on exoskeleton usability, and product design research converge on a clear point: setup and takedown tasks are highly susceptible to both usability problems and product design-driven complexities. Yet despite this broad evidence base, there has been little systematic investigation of how these factors interact in the exoskeleton context where shared use, repetitive setup and takedown, and task-specific customization are common. Most importantly, no investigations have examined the complexities of the setup and takedown tasks for exoskeletons and how they could impact worker performance metrics.

Task complexity theory in human factors engineering provides a useful framework to examine the complexities arising from usability problems and product design architecture and how they impact exoskeleton setup and takedown tasks [59]. Task complexity can be viewed from structuralist, resource requirement, and interaction perspectives. The structuralist perspective focuses on the elements in the task structure such as the task outcomes, actions, and information cues. The resource requirements perspective defines complexity in terms of the physical and cognitive resources required for successful task completion. The interaction perspective considers how task complexity arises from the interaction between the task and the task performer, including prior knowledge and experience of the worker. Liu & Li [59] identified key task complexity dimensions, including task size (number of task components), variety, ambiguity, interdependencies and relationships, variability, unreliability measures, novelty, inconsistencies, action complexities, and temporal demands. They recommend selecting task dimensions most relevant to the specific application. In the exoskeleton context, the most relevant dimensions include the number of task steps, the time required for setup and takedown tasks, and the physical and cognitive requirements of human actions arising from usability barriers, and the part count influencing the structural complexity of the task.

Building on these insights, we hypothesize that task complexity, operationalized through the number of task steps, usability problems, and part count, significantly influences task completion time and success rates. We test this hypothesis using an experimental framework that systematically analyzes setup and takedown tasks across multiple exoskeleton designs to isolate the role of specific task complexity dimensions.

Our study is guided by three research questions: (1) How much time do users spend on setup (assembly, donning) and takedown (doffing, disassembly) tasks, and how successful are they in completing these tasks? (2) How do task complexity factors – number of steps in the task, the usability problems, and the part count predict setup and takedown time and task success? and (3) Which of these factors best inform design strategies for improving deployment? By addressing these questions, we investigate whether setup and takedown procedures pose barriers to exoskeleton deployment and identify specific design factors that can inform real-world exoskeleton deployment.

This study makes several novel contributions to the exoskeleton literature. First, to our knowledge, this is the first systematic investigation of setup and takedown procedures as barriers to exoskeleton deployment. While prior research has extensively examined biomechanical and physiological outcomes during exoskeleton use, the tasks required before and after use, which directly determine whether workers can independently and reliably deploy these devices, have received little empirical attention. Second, our findings challenge the intuitive assumption that physical simplicity (fewer parts) translates to ease of use; we demonstrate that procedural complexity, operationalized through hierarchical task analysis, is the dominant predictor of both task completion time (R^2^ = 0.753) and assembly failure rates (R^2^ = 0.968), while part count shows no significant relationship with either outcome. Third, we identify differential complexity drivers across setup phases: assembly performance is primarily determined by the number of procedural steps, whereas donning performance is more strongly associated with usability problems. This distinction has not been previously documented and suggests that design optimization strategies should be tailored to specific deployment phases. These findings extend task complexity theory to the exoskeleton domain and provide actionable design guidance for improving real-world deployability.

## 2. Methods

### 2.1. Study design

We conducted a controlled laboratory study using a within-subjects design to quantify time demands and task completion rates during setup (assembly and donning) and takedown (doffing and disassembly) tasks across four occupational exoskeletons. Each participant performed all four tasks with each of the four exoskeletons, resulting in 16 task-exoskeleton combinations per participant. The study protocol was approved by the Institutional Review Board for Human Subjects Research at the University of Texas at El Paso (protocol #2137912−2), and all participants provided written informed consent prior to participation. All experiments were conducted in the Physical, Information and Cognitive Human Factors Engineering (PIC-HFE) Research Laboratory at the University of Texas at El Paso.

### 2.2. Study participants

We recruited twenty-nine participants (15 males, 14 females) from two undergraduate engineering classes at the University of Texas at El Paso. All participants were senior engineering students, with a mean age of 23.4 years (SD = 3.5). To minimize potential coercion bias, instructors emphasized that participation was voluntary and that alternative projects were available for students who chose not to participate. To be eligible for the study, participants were required to have no history of musculoskeletal disorders or injuries within the past year. Participants had technical problem-solving experience from their undergraduate coursework but otherwise had no prior training in exoskeleton assembly or operation, making them representative of technically capable but exoskeleton-naïve workers who might encounter these devices in industrial settings.

### 2.3. Devices and materials

#### 2.3.1. Exoskeletons Used.

We used four occupational exoskeletons in the study: the Ironhand, Chairless Chair, Skelex, and Laevo. These exoskeletons were selected based on their market availability, differences in their intended industrial applications, and diversity of design approaches. The four exoskeletons were selected to represent the diversity of occupational exoskeleton designs currently available for industrial deployment, spanning different body regions (hand, upper limb, trunk, and lower limb) and assistance mechanisms (powered and passive). This selection reflects the practical reality that organizations evaluating exoskeleton adoption must often compare devices across functional categories based on specific work demands. The key design characteristics of each device are presented in Table 1.

**Table 1:** Key elements of the four exoskeletons tested in this study, including potential limiting factors for setup and takedown tasks.

Exoskeleton name and type	Functional domain	Key setup and takedown requirements	Key device characteristics	Key limiting factors for ease of setup and takedown tasks
Ironhand (powered grip-assisting glove)	Hand and forearm support	• Connect glove modules • Attach to power unit • Route cables • Secure straps	• Highly modular: separate battery, power pack, glove connector, and backpack/hip carrying device • Clear visual cues with battery indicator lights (green/orange) and grooved surfaces for grips • Intuitive connections with magnetic attachments, click mechanisms, and obvious orientation cues • Simple assembly logic with a linear sequence and clear decision points • User-friendly features such as Velcro adjustments, cord clips, and remote control holders	Minimal: intuitive connections eliminate assembly complexity
Chairless Chair (lower-limb sit/stand support)	Postural support	• Adjust leg length • Secure thigh straps • Lock joints into correct angles	• Streamlined components including a main frame, sliders, rubber feet and belt systems • Obvious mechanical cues including orange levers, magnetic liders, and size adjustment points with visible markings • Height-based sizing with simple measurement-to-setting correspondence • Minimal sub-assembly, with the most complexity in belt attachment and sizing	Moderate: leg support devices require some inherent body positioning complexity
Skelex (passive shoulder support device)	Overhead arm support	• Align shoulder frames • Attach straps • Tension arm supports	• Fit-oriented engineering with multiple, measurement-dependent adjustment points (waist, height, arm length, and weight) • Sequential component integration, from belt connectors in a flex frame, to Minax, to arm cups • Multiple sizing decisions including arm circumference, force settings, and cup sizes • Precision requirements including 180-degree rotations, specific window alignments, and force adjustments	High: measurement requirements, technical procedures, manual strength requirements
Laevo (passive trunk support device)	Back and torso support	• Take six body measurements • Adjust torsion springs • Fit torso and shoulder straps	• High setup complexity: requires six different measurements, sizing form completion, and multiple component sizes • Precision assembly that requires hex keys, specific torque settings, and careful alignment procedures • Component interdependence: vest frame padding, front vest, back connector, and torso structures depend on each other • Multiple adjustment cycles with length settings, angle changes, and fit checks	Very high: tool requirements, extensive measurements, and two-person operation

**Fig 1:**
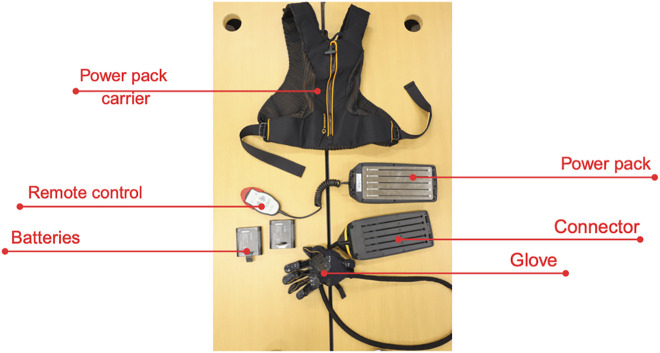
Parts and components of the Ironhand exoskeleton.

The Ironhand (Bioservo Technologies AB, Sweden) is a soft, active hand exoskeleton designed to augment grip strength during manual handling tasks. The system consists of a glove unit with artificial tendons, a power pack with a battery and control unit, and a remote control for adjustments. The power pack can be worn in either a backpack or a hip-belt configuration (Fig 1).

**Fig 2:**
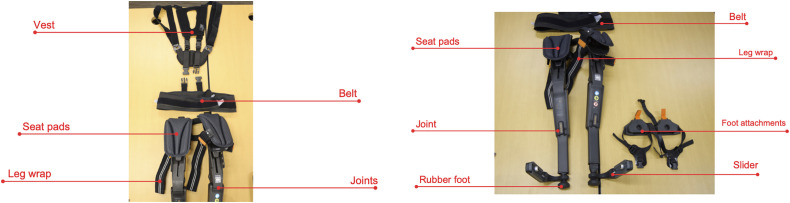
Parts and components of the Chairless Chair exoskeleton.

The Chairless Chair (Noonee AG, Switzerland) is a passive lower-limb exoskeleton designed to provide support during prolonged standing and squatting. The device consists of a frame with adjustable struts, thigh and calf supports, and a belt system for securing the device to the body (Fig 2).

**Fig 3:**
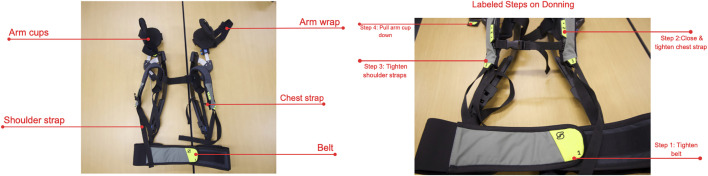
Parts and components of the Skelex exoskeleton.

The Skelex 360-XFR (Skelex BV, The Netherlands) is a passive upper-limb exoskeleton designed to reduce shoulder load during overhead work. The system uses a spring-based mechanism to provide arm support, with adjustable force settings and modular arm cups attached to a back frame (Fig 3).

**Fig 4:**
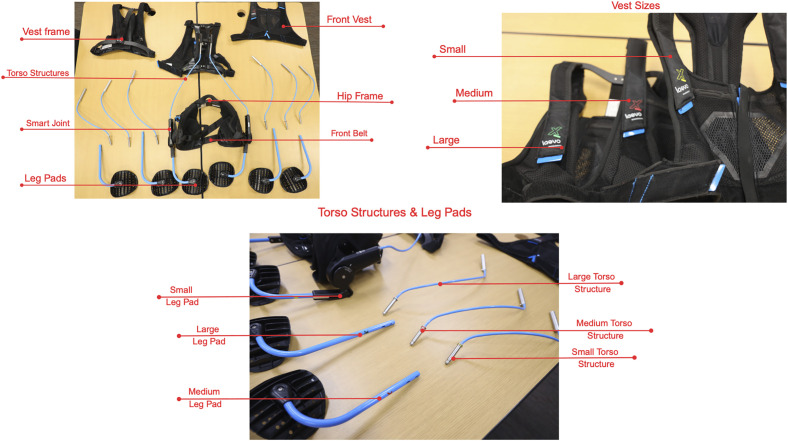
Parts and components of the Laevo exoskeleton.

The Laevo (Laevo BV, The Netherlands) is a passive exoskeleton designed to reduce lower back load during forward bending and lifting tasks. The device uses springs to transfer load from the back to the legs through a chest pad and thigh pads connected by a rigid frame (Fig 4).

#### 2.3.2. Other equipment and materials.

To record the start and stop times for each task for each participant and exoskeleton, we built a custom macro in Microsoft Excel with “start” and “stop” buttons for each task phase. The macro also recorded task completion (i.e., success or failure). Participants performed all setup and takedown tasks from a standard worktable where the exoskeleton parts and materials were placed. All required tools, including measuring tapes and hex keys, were supplied by the manufacturer; no additional tools were provided to the participants. Printed and online manufacturer-provided instruction manuals were also available.

### 2.4. Procedure

Participants completed the study individually in a single two-hour session, with 30 minutes allocated to each exoskeleton. Two investigators were present during each experimental session. Participants were instructed to wear thin, loose-fitting clothing for the experimental session. When they arrived, participants received an overview of the study objectives, safety procedures, and task requirements, and were informed that they had a maximum of 30 minutes for the complete setup and takedown of each exoskeleton. They were told that they could use the printed or online instruction manuals for guidance or ask an investigator for clarification if they could not complete a task step using the provided instructions. However, no verbal or physical assistance was provided unless participants explicitly requested help or safety concerns arose. Participants were encouraged to work efficiently and problem-solve independently before requesting assistance. Participants were also informed that they could stop at any time if they felt they could not continue with a task. The order in which exoskeletons were presented was randomized across participants to control for any order and fatigue effects, and between each exoskeleton, participants took a 10-minute break.

For each exoskeleton, participants completed the following sequence of four tasks:

a **Assembly:** Participants were given the disassembled exoskeleton components (as received from the manufacturer) and instructed to assemble the device as quickly and accurately as possible following the manufacturer’s instructions.
b **Donning:** Following assembly, participants were instructed to don the exoskeleton as quickly and accurately as possible, making all adjustments for proper fit.
c **Doffing:** After donning was complete, participants were instructed to remove the device while ensuring that all straps were properly released and all components safely removed.
d **Disassembly:** Participants were instructed to disassemble the exoskeleton and return it to its storage configuration.

### 2.5. Measurements

#### 2.5.1. Primary outcome measures.

We measured two primary outcomes for each task:

a **Task completion time**, measured as the time in minutes from task initiation (first contact with the device components) to task completion (successful achievement of the manufacturer-defined end state).
b **Task completion rate**, recorded as a binary measure (success or failure) for each task. Success was defined as achieving the correct manufacturer-specified end configuration. Failure was recorded if a participant reported that they could not complete the task within the allocated 30-minute time.

#### 2.5.2. Explanatory variables.

As defined above, we considered three different measures of task complexity that may have affected task completion times and rates.

**Number of steps or task size:** Prior to participant testing, three authors conducted a comprehensive hierarchical task analysis (HTA) for each exoskeleton. The HTA was conducted using a consensus-based methodology involving three trained analysts. Each analyst independently reviewed manufacturer documentation and instructional materials for all four exoskeletons, then developed preliminary task decompositions following established HTA guidelines. The team then convened in five structured consensus meetings to reconcile differences, discuss decomposition logic, and establish consistent granularity across devices. Disagreements were resolved through discussion until unanimous agreement was reached on the final step counts. This consensus approach prioritized methodological consistency across devices over independent coding, as our primary goal was to establish comparable complexity metrics rather than assess inter-rater reliability per se. We acknowledge that HTA granularity is inherently analyst-dependent; our decomposition aimed for a level of detail capturing meaningful user actions (e.g., ‘attach strap to buckle’) rather than micro-movements (e.g., ‘grasp strap end’). The manufacturer documentation served as the primary reference to ensure that the identified steps reflected intended procedures. The final HTA with each task decomposed into discrete, observable steps is shown in Supporting Information S1 File.

**Usability problems:** As part of a separate study prior to participant testing [60], a structured heuristic usability assessment was conducted for each exoskeleton, combining Nielsen’s heuristics with Shneiderman’s golden rules for interface design. For this study, seven expert evaluators independently identified usability problems for each task phase for each exoskeleton and scored them on a severity scale (0 = not a problem, 1 = cosmetic, 2 = minor, 3 = major, and 4 = catastrophic). Individual ratings were then reconciled into a comprehensive list of usability problems for each task-exoskeleton combination, and the total number of usability problems for each task-exoskeleton combination was used as our measure of usability in this study. Problems were included regardless of their severity rating to capture the full range of potential difficulties.

**Part count:** As a third predictor, we counted the total number of separate physical components in each exoskeleton. These included the structural components, straps, fasteners, adjustment mechanisms, and removable padding or accessories that users must manipulate during exoskeleton setup and takedown.

### 2.6. Data analysis

All statistical analyses were performed using R Version 4.5.1. Statistical significance was set at α = 0.05 for all analyses, and effect sizes were interpreted according to Cohen’s conventions (small: d = 0.2, medium: d = 0.5, large: d = 0.8).

#### 2.6.1. Descriptive statistics.

We calculated the means and standard deviations for task completion times across all task-exoskeleton combinations. Task completion rates were calculated as the percentage of successful completions from the total sample (n = 29) for each task-exoskeleton combination.

#### 2.6.2. Inferential statistics.

We used three separate approaches to test for variation in task completion times and rates as a function of our different experimental and explanatory variables.

**Effects of task and exoskeleton type:** We assessed effects of task and exoskeleton type using separate one-way repeated measures ANOVAs, with participant as the repeated factor. For the task effect, completion times were averaged within each participant across exoskeletons before analysis; for the exoskeleton effect, times were averaged across tasks. This approach allowed inclusion of all 29 participants while properly accounting for the within-subjects design. Mauchly’s test was used to assess sphericity, with Greenhouse-Geisser corrections applied when violated. Partial eta-squared (η²p) was calculated as the effect size measure. Post-hoc pairwise comparisons were conducted using Bonferroni correction.

**Effect sizes:** To assess the practical significance of the differences in task completion times, we calculated Cohen’s d for each pairwise comparison between exoskeletons.

**Design-level regression analyses:** Finally, we used linear regression to more rigorously test for relationships between our explanatory variables for task complexity (number of steps, usability problems, and part count) and outcome measures (task completion time and success rate). We performed a single pooled analysis across all 16 task-exoskeleton combinations and separate task-specific analyses. The proportion of variance explained by each predictor was assessed using the coefficient of determination (R^2^). Simple linear regression models of the form Y = β₀ + β₁X + ε were fitted, where Y represents the outcome variable (task completion time in minutes or failure rate as percentage), β₀ is the intercept, β₁ is the regression coefficient for predictor X, and ε represents the residual error assumed to be normally distributed with mean zero. Three separate models were fitted for each outcome variable: one with HTA steps as the predictor, a second with number of usability problems, and the third with part count. Model fit was assessed using the F-statistic and associated p-value, with statistical significance set at α = 0.05. Regression coefficients, standard errors, and 95% confidence intervals were computed to characterize the magnitude and precision of predictor effects. Model assumptions including linearity, homoscedasticity, and normality of residuals were verified through visual inspection of diagnostic plots and Shapiro-Wilk tests.

**Participant-level and order effects analyses:** To complement the design-level regression analyses described above, we conducted participant-level regression analyses using all individual observations (N = 397; 29 participants × up to 16 task-exoskeleton combinations per participant, with some combinations missing due to task non-completion). This approach provides greater statistical power than design-level analyses while utilizing all available data. We also computed within-participant correlations between complexity metrics and completion time to confirm that relationships held within individuals, not merely between experimental conditions. To assess potential learning effects across the testing session, we examined whether exoskeleton presentation order predicted completion time and computed partial correlations between complexity metrics and completion time controlling for presentation order.

## 3. Results

We evaluated time to completion and overall success rate for two setup tasks (assembly and donning) and two takedown tasks (doffing and disassembly) across four different occupational exoskeletons. We then analyzed these outcomes in relation to task complexity explained by three key variables for each exoskeleton and task: (1) number of steps, (2) usability problems, and (3) part count.

### 3.1. Setup tasks consumed the most time, had more steps and usability problems, and exhibited substantial variability across exoskeletons

Table 2 presents summary statistics for the completion time and success rate for the different setup and takedown tasks for each exoskeleton. Our findings indicate that setup tasks (i.e., assembly and donning) accounted for a significant portion of the 30-minute time window allotted for each exoskeleton, ranging from one-third of the window for Ironhand to nearly 27 minutes for Laevo. Assembly alone required more than 15 minutes for the Skelex and Laevo exoskeletons. In contrast, takedown tasks (i.e., doffing and disassembly) required less time (Table 2), likely reflecting participants’ increased familiarity with the device following their completion of the setup tasks.

**Table 2:** Task performance measures and design characteristics of exoskeletons.

Exoskeleton *Setup & Takedown Tasks*	Task and design measures				
	Average time (minutes)	Completion rates (%)	Number of HTA steps	Number of usability problems	Part count
Ironhand			55 steps total	34 problems total	20
*Assembly*	6.5	100	25	10	
*Donning*	3.6	100	14	9	
*Doffing*	0.95	100	9	5	
*Disassembly*	1.8	96.6	7	10	
Chairless Chair			77 steps total	51 problems total	22
*Assembly*	9.8	96.6	35	18	
*Donning*	6.3	96.6	22	18	
*Doffing*	0.8	96.6	10	11	
*Disassembly*	1.1	96.6	10	4	
Skelex			73 steps total	66 problems total	5
*Assembly*	15.5	79.3	45	26	
*Donning*	5.3	65.5	16	18	
*Doffing*	0.56	65.5	6	11	
*Disassembly*	1.6	65.5	6	11	
Laevo			136 steps total	41 problems total	22
*Assembly*	24.6	51.7	65	14	
*Donning*	2.8	51.7	47	10	
*Doffing*	0.48	48.3	6	6	
*Disassembly*	4.0	48.3	18	11	

The number of steps required for setup tasks was also markedly higher than for the takedown tasks. Based on our HTA, the number of procedural steps was highest for the assembly task, ranging from 25 steps for the Ironhand to 65 steps for the Laevo (Table 2). Together with the donning task, a user must complete anywhere from 39 steps for the simplest setup (Ironhand) to 112 steps for the most complex setup (Laevo), likely incurring a substantial cognitive and physical demand on the user. We also identified 123 usability problems [60] for setup tasks, the highest among all tasks, indicating that not only the number of steps involved, but also the ease of procedure execution affects a user’s ability to successfully complete a setup task efficiently and without failure. Part counts did not seem to impact completion times or rates, indicating that the number of physical components may be less important than the number of steps and usability.

### 3.2. Assembling the exoskeleton emerged as the most time-intensive task

**Fig 5:**
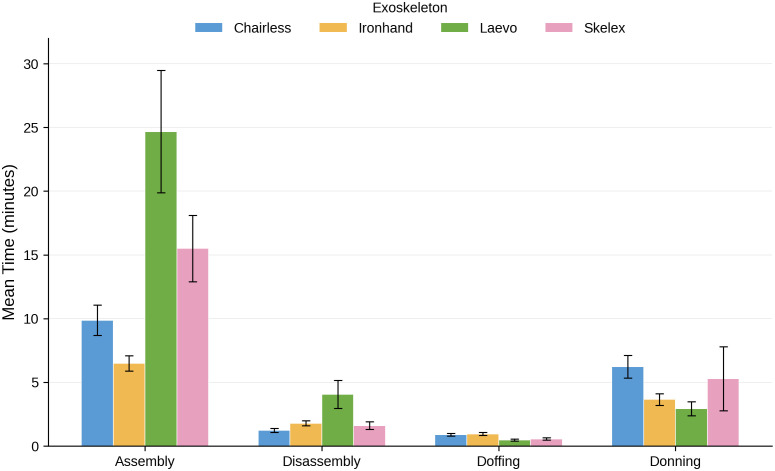
Mean task completion times for each task and exoskeleton type..

One-way repeated measures ANOVA confirmed that task type had a significant effect on completion time (F(3, 84) = 197.77, p < .001, η²p = 0.88), with assembly taking substantially longer than other tasks. A separate repeated measures ANOVA confirmed a significant main effect of exoskeleton type (F(3, 84) = 27.47, p < .001, η²p = 0.50), indicating significant differences in completion times across devices. Overall, assembly task also exhibited the most variability in completion times, ranging by almost four-fold from 6.5 minutes for the Ironhand to 24.6 minutes for the Laevo (Fig 5).

**Fig 6:**
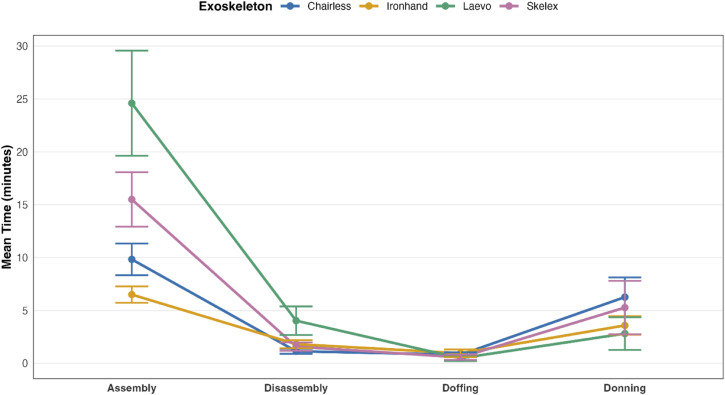
Interaction effect between task and exoskeleton type as predictors of task completion time.

For the 11 participants who completed all 16 task-exoskeleton combinations, a two-way repeated measures ANOVA revealed a significant Task × Exoskeleton interaction (F(9, 90) = 15.60, p < .001, η²p = 0.61), indicating that exoskeleton performance varies depending on the specific task being performed (Fig 6). Specifically, some exoskeletons (e.g., Laevo) imposed greater time burdens in specific tasks (notably assembly and disassembly), whereas other devices (e.g., Ironhand) had consistently low completion times across all task phases.

**Fig 7:**
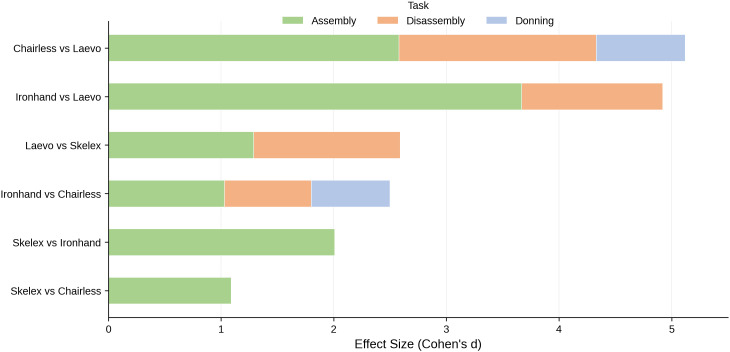
Effect sizes for pairwise comparisons between exoskeletons; only statistically significant differences (p < 0.05) shown. **Large effects (green bars) for assembly tasks dominate the comparisons.**.

Pairwise comparisons between exoskeletons further highlighted the higher burden of the assembly tasks. Of all the setup and takedown tasks, exoskeleton assembly demonstrated the most substantial effect sizes in completion time, ranging from large to very large (Cohen’s d = 1.03–3.67). The largest difference in assembly time was between the Ironhand and Laevo (d = 3.67), reflecting a practical difference in how these devices are designed to be assembled for deployment (Fig 7). In contrast, disassembly showed only moderate to large effect sizes (d = 0.77–1.75), with the Laevo consistently requiring more disassembly time than other devices. Doffing times for each exoskeleton were minimal compared to their assembly and donning times.

### 3.3. Design-level associations between complexity metrics and task completion time

We used linear regression to model completion times for each task as a function of number of steps from HTA, the number of usability problems, and the part counts for each exoskeleton. We first conducted design-level analyses examining how these complexity metrics related to mean completion times across the 16 exoskeleton-task combinations (4 exoskeletons × 4 tasks). Each data point in these analyses represents the mean completion time for a specific exoskeleton-task combination.

**Fig 8:**
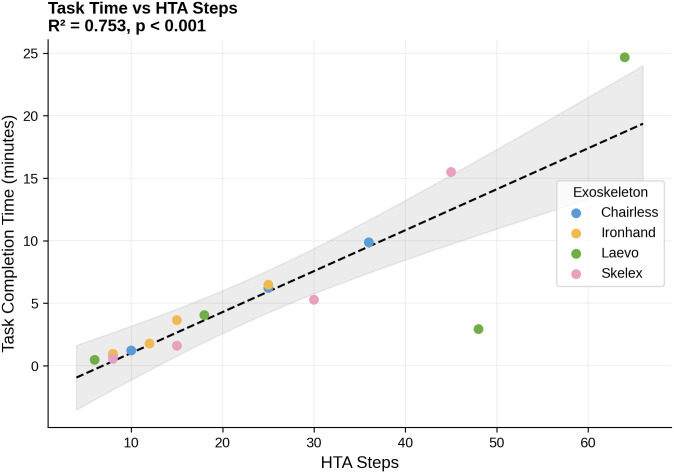
Task completion times as a function of number of HTA steps for the 16 task-exoskeleton combinations tested here (four tasks, four devices).

**Fig 9:**
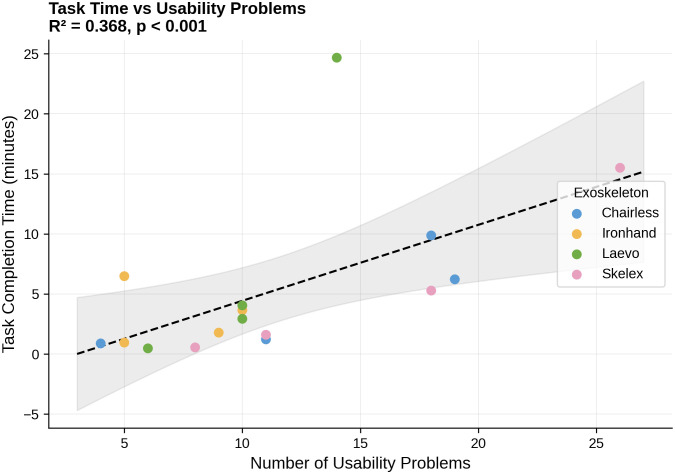
Task completion times as a function of the number of usability problems for the 16 task-exoskeleton combinations tested here (four tasks, four devices).

Results from the design-level regression analysis showed that number of HTA steps was the strongest predictor of mean completion time (β₁ = 0.33, SE = 0.05, 95% CI [0.22, 0.44]), accounting for 75.3% of variance across the 16 design conditions (F(1,14) = 42.76, p < .001). This coefficient indicates that each additional procedural step is associated with an average increase of approximately 20 seconds in task completion time (Fig 8). The number of usability problems was also a significant predictor (β₁ = 0.62, SE = 0.18, 95% CI [0.24, 1.00]), accounting for 47.4% of variance (F(1,14) = 12.63, p = .003), with each additional usability problem associated with approximately 37 seconds of additional task time (Fig 9). In contrast, part count showed no significant relationship with completion time (β₁ = 0.09, SE = 0.31, 95% CI [−0.58, 0.76], p = .779, R² = 0.006). Complete regression statistics are presented in Table 3.

**Table 3:** Pooled Regression Analysis: Task Completion Time (N = 16).

Predictor	β₀ (SE)	β₁ (SE)	R²	F(1,14)	p	95% CI (β₁)
HTA Steps	−2.84 (1.89)	0.33 (0.05)	0.753	42.76	<.001***	[0.22, 0.44]
Usability Problems	−3.21 (2.98)	0.62 (0.18)	0.474	12.63	.003**	[0.24, 1.00]
Part Count	5.12 (5.41)	0.09 (0.31)	0.006	0.08	.779	[-0.58, 0.76]

Note. SE = Standard Error; CI = Confidence Interval. **p <.01, ***p <.001.

We conducted exploratory task-specific analyses to examine whether complexity-performance associations differed across task phases. These analyses should be interpreted with caution given the limited number of design conditions (N = 4 exoskeletons per task), which increases the risk of unstable parameter estimates.

**Fig 10:**
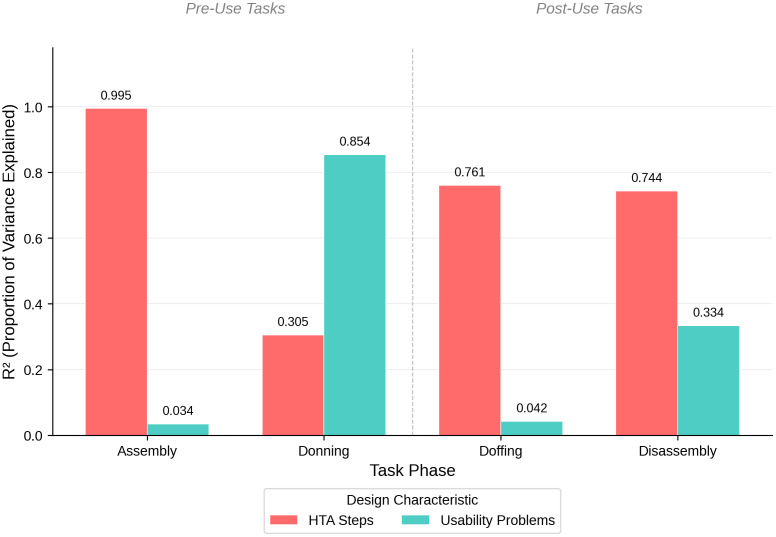
Proportion of variance in task completion times (R^2^) explained by number of HTA steps (procedural complexity) and number of usability problems across setup and takedown tasks.

At the design level, assembly task completion times showed the strongest correlation with number of steps (R² = 0.995, p = 0.003), while donning times trended toward a stronger correlation with usability problems (R² = 0.854, p = 0.076) than with number of steps (Fig 10). Takedown tasks (doffing and disassembly) showed moderate correlations with number of steps (R² = 0.761 and 0.744, respectively), though these were not statistically significant given the small sample size. These exploratory patterns suggest that different complexity factors may predominate in different deployment phases, though confirmation with larger samples is warranted. Complete task-specific statistics are presented in Table 4.

**Table 4:** Task-specific regression analyses: task completion time.

Task	Predictor	β₁ (SE)	R²	F(1,2)	p	Interpretation
Assembly	HTA Steps	0.45 (0.02)	0.995	374.8	.003**	Very Strong
	Usability Problems	0.28 (1.08)	0.034	0.07	.815	Not Significant
Donning	HTA Steps	0.08 (0.09)	0.305	0.88	.447	Not Significant
	Usability Problems	0.39 (0.10)	0.854	11.68	.076†	Trending
Doffing	HTA Steps	0.12 (0.04)	0.761	6.38	.127	Moderate
Disassembly	HTA Steps	0.22 (0.08)	0.744	5.80	.138	Moderate

Note. †p < .10, **p < .01. N = 4 exoskeletons per task; results should be interpreted with caution due to small sample size.

**Fig 11:**
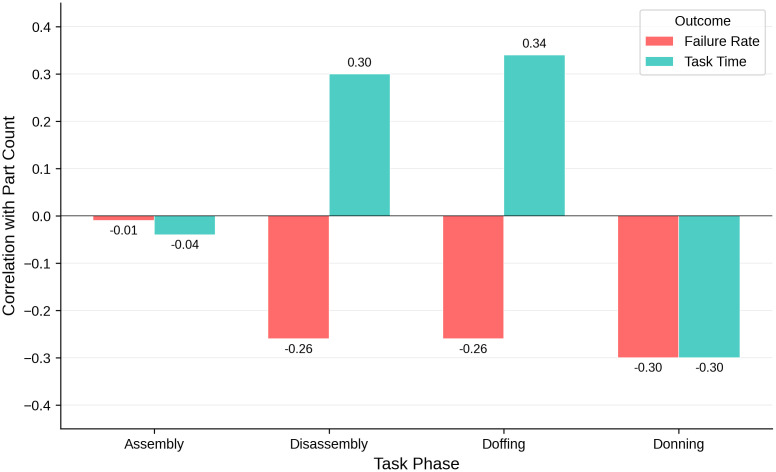
Weak correlations between part count and each of completion time and failure rate for the different setup and takedown tasks.

Part counts for each exoskeleton exhibited little to no correlation with completion times for all four tasks, with correlation coefficients ranging from r = −0.01 to 0.30 (Fig 11). This finding illustrates that a device can be physically simple but still present challenges for the user: for example, the Skelex system had only five parts yet completion times ranged from about one minute to nearly 15 minutes across tasks. In contrast, the Ironhand had 20 parts but all four tasks could be completed more quickly. The Laevo and Chairless Chair each had 22 parts but differed markedly in setup and takedown times.

### 3.4. Design-level associations between complexity metrics and task failure rates

**Fig 12:**
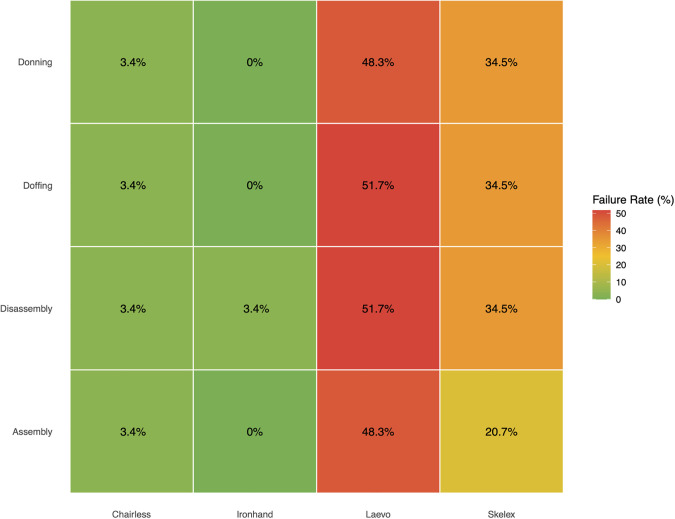
Task failure rates for different exoskeletons.

Overall failure rates (Fig 12) ranged from 0% for Ironhand to 50% for Laevo. All 29 participants successfully assembled and donned the Ironhand, whereas only 15 successfully assembled and donned the Laevo. The Skelex performed marginally better than the Laevo, with 23 participants successfully assembling it and 19 completing the other tasks. Only one participant could not assemble the Chairless Chair.

**Fig 13:**
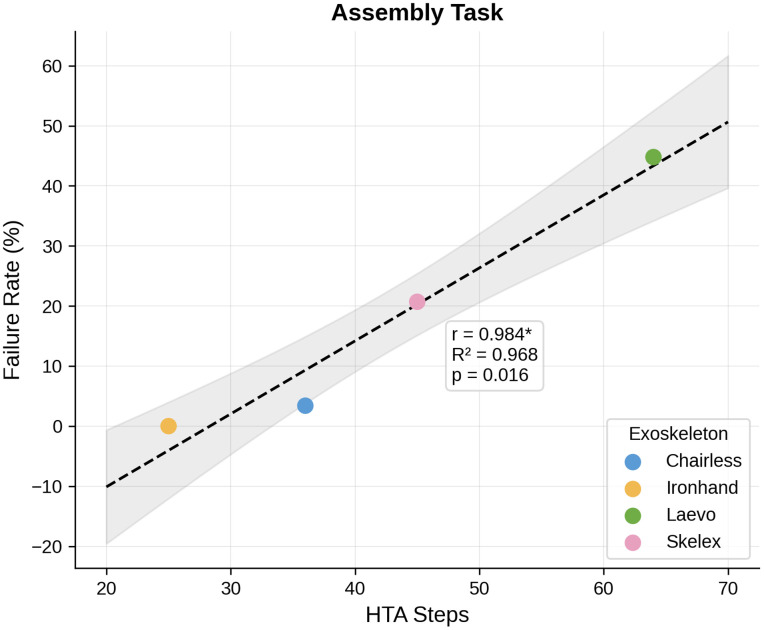
Task failure rates as a function of the number of HTA steps for the assembly task.

We evaluated how number of steps, usability problems, and part counts were associated with task failure rates (Fig 13). Design-level regression across the 16 task-exoskeleton combinations revealed that failure rates showed weak associations with number of steps (R² = 0.076, p = 0.302), usability problems (R² = 0.001, p = 0.888), and part counts (R² = 0.045, p = 0.460), indicating that these design elements did not have a uniform effect on failure rates across all tasks.

However, exploratory task-specific analyses revealed that assembly failure rates showed a strong association with number of steps (R² = 0.968, p = 0.016), with rates ranging from 0% (Ironhand, 25 HTA steps) to 48.3% (Laevo, 65 HTA steps) (Fig 13). This pattern suggests that the number of procedural steps for assembly may be associated with whether users can successfully complete the task, though this finding should be interpreted cautiously given the small sample of devices (N = 4). Doffing failure rates showed a trending association with number of steps (R² = 0.861, p = 0.076), while donning and disassembly failure rates showed no significant relationships with any complexity metric (Table 5).

**Table 5:** Regression analysis: task failure rates.

Analysis	Predictor	β₁ (SE)	R²	F	p	95% CI
Pooled (N = 16)	HTA Steps	0.21 (0.19)	0.076	1.15	.302	[-0.20, 0.62]
	Usability Problems	−0.04 (0.73)	0.001	0.02	.888	[-1.59, 1.51]
	Part Count	0.86 (1.13)	0.045	0.57	.460	[-1.54, 3.26]
Assembly (N = 4)	HTA Steps	1.19 (0.11)	0.968	60.51	.016*	[0.72, 1.66]
	Usability Problems	0.33 (2.35)	0.010	0.02	.901	[-9.80, 10.46]

Note. *p < .05. Failure rate expressed as percentage (0–100).

Table 6 summarizes the design-level regression results, including the model, the fitted equations, the associated R^2^ values and the p-values for the models.

**Table 6:** Summary of fitted design-level regression models.

Model	Fitted Equation	R²	p
Pooled: Time ~ HTA	Time = −2.84 + 0.33 × HTA Steps	0.753	<.001***
Pooled: Time ~ Usability	Time = −3.21 + 0.62 × Usability Problems	0.474	.003**
Assembly: Time ~ HTA	Time = −4.72 + 0.45 × HTA Steps	0.995	.003**
Donning: Time ~ Usability	Time = −1.86 + 0.39 × Usability Problems	0.854	.076†
Assembly: Failure ~ HTA	Failure Rate = −29.52 + 1.19 × HTA Steps	0.968	.016*

Note 1. Time in minutes; Failure Rate in percentage. †p < .10, *p < .05, **p < .01, ***p < .001.

Note 2. The following is the interpretation of the regression coefficients: (1) **HTA Steps (Pooled):** β₁ = 0.33 indicates that each additional procedural step is associated with an increase of 0.33 minutes (approximately 20 seconds) in task completion time, holding other factors constant; (2) **Usability Problems (Pooled):** β₁ = 0.62 indicates that each additional usability problem is associated with an increase of 0.62 minutes (approximately 37 seconds) in task completion time; (3) **Assembly Failure Rate:** β₁ = 1.19 indicates that each additional HTA step in assembly is associated with a 1.19 percentage point increase in failure rate.

### 3.5. Participant-level regression analyses

To provide a more robust test of the complexity-performance relationships while utilizing all available data, we conducted participant-level regression analyses using all 397 individual observations (29 participants × up to 16 task-exoskeleton combinations per participant, with some combinations missing due to task non-completion). Results are presented in Table 7.

**Table 7:** Participant-level regression results: Predictors of task completion time (N = 397 observations).

Predictor	β	SE	95% CI	p	R²
HTA Steps	0.374	0.013	[0.348, 0.400]	<.001***	0.669
Usability Problems	0.720	0.055	[0.613, 0.828]	<.001***	0.305
Part Count	−0.084	0.056	[-0.194, 0.025]	.133	0.006

Note. Each predictor tested in separate model. β = unstandardized regression coefficient representing minutes per unit increase in predictor. ***p < .001*.*

Participant-level analyses confirmed the associations observed at the design level. HTA step count was a significant predictor of completion time (β = 0.374, SE = 0.013, 95% CI [0.348, 0.400], p < .001), explaining 66.9% of variance. Each additional procedural step was associated with approximately 22 seconds longer completion time. The number of usability problems also significantly predicted completion time (β = 0.720, SE = 0.055, 95% CI [0.613, 0.828], p < .001, R² = 0.305), while part count showed no significant association (β = −0.084, SE = 0.056, 95% CI [−0.194, 0.025], p = .133, R² = 0.006).

To confirm that the complexity-performance relationship held within individuals rather than merely reflecting between-condition differences, we computed within-participant correlations between HTA steps and completion time for each participant. The average within-participant correlation was r = 0.850 (SD = 0.109), and a one-sample t-test confirmed this was significantly different from zero (t(28) = 41.20, p < .001). This indicates that the relationship between procedural complexity and completion time holds robustly within individuals across different exoskeleton-task combinations.

### 3.6. Order effects analysis

To assess whether the complexity-performance associations might be confounded by learning effects across the testing session, we examined whether exoskeleton presentation order predicted completion time. If participants improved through general skill transfer across devices, later-presented exoskeletons would show faster completion times regardless of their complexity.

Order effects analysis revealed no significant learning effect across devices (β = −0.395 minutes per position, SE = 0.349, p = .258). Although the negative coefficient suggests a trend toward faster completion with later-presented devices, this effect did not reach statistical significance. Critically, the relationship between HTA steps and completion time remained highly significant after controlling for presentation order (partial r = 0.814, p < .001), indicating that the complexity-performance association is not an artifact of cross-device learning.

Task-specific order effects were also examined. Assembly showed the largest (though non-significant) order effect (β = −1.065 minutes per position, p = .140), suggesting some procedural learning may transfer across devices for this task. Donning (β = 0.115, p = .801), doffing (β = −0.025, p = .721), and disassembly (β = 0.043, p = .787) showed minimal order effects.

In summary, both design-level and participant-level analyses converge on the finding that procedural complexity, operationalized as HTA step count, is the strongest predictor of setup and takedown completion time. This association is robust to controls for presentation order and holds within individuals across different exoskeleton-task combinations. Part count, in contrast, shows no significant relationship with performance at either level of analysis.

## 4. Discussion

Our study provides the first systematic evidence of task complexity in exoskeleton setup and takedown procedures and their significant impact on time and completion rates – identifying a potential barrier to industrial deployment. We found that task complexity influenced task completion time and success, but its component factors (number of steps, usability problems and part count) had differing impacts. The number of steps required to complete a task emerged as the strongest predictor of task completion time and success for all setup and takedown tasks. Usability problems played a secondary role, particularly during donning, while part count had little explanatory power. We additionally found that the setup tasks, especially exoskeleton assembly, represent a more substantial barrier than the other factors that previous studies have considered [2,61,62]. For example, our finding that some exoskeletons required nearly 30 minutes to assemble challenges current assumptions about the practical viability of exoskeletons in time-sensitive industrial environments. These results suggest that exoskeleton design may benefit from reducing the number of task steps rather than focusing solely on usability fixes or minimizing part count, with a critical focus on setup tasks (see Table 8 relating key findings with the statistical evidence, and the practical implications of the finding).

**Table 8:** Key findings summary with statistical evidence.

Key Finding	Statistical Evidence	Practical Implication
Procedural complexity is the primary predictor of task time	Design-level: β = 0.33, R² = 0.753, p < .001; Participant-level: β = 0.374, R² = 0.669, p < .001; Within-participant r = 0.850	Each additional HTA step adds ~20 seconds to task time
Part count does not predict performance	Design-level: R² = 0.006, p = .779; Participant-level: R² = 0.006, p = .133	Physical simplicity ≠ ease of use
Assembly failure strongly linked to HTA steps	R² = 0.968, p = .016 ((N = 4; interpret with caution)	Reducing procedural steps may improve for task success
Donning shows stronger usability association	Usability: R² = 0.854, p = 0.076; HTA: R² = 0.305, p = .447	Different optimization strategies may be needed for different phases
Assembly is the primary time burden	Range: 6.5–24.6 min; Cohen’s d = 1.03–3.67	Design efforts should prioritize assembly simplification
No significant order/learning effects	Order: β = −0.395, p = .258 HTA | Order: partial r = 0.814, p < .001	Complexity-performance relationship not confounded by learning

Note. HTA = Hierarchical Task Analysis. Design-level analyses: N = 16 exoskeleton-task combinations. Participant-level analyses: N = 397 individual observations. Task-specific design-level analyses (N = 4) should be interpreted with caution due to small sample size.

The robustness of our findings is supported by convergent evidence across multiple analytic approaches. Our design-level analyses (N = 16 exoskeleton-task combinations) identified procedural step count as the strongest predictor of completion time (R² = 0.753), and participant-level analyses using all 397 individual observations confirmed this association with a more conservative estimate (R² = 0.669, β = 0.374, p < .001). Importantly, within-participant correlations averaged r = 0.850, indicating that the complexity-performance relationship holds within individuals across different tasks and devices, not merely as a between-condition artifact. Furthermore, order effects analysis revealed no significant cross-device learning (p = .258), and the HTA-time relationship remained robust after controlling for presentation order (partial r = 0.814, p < .001). Together, these findings suggest that procedural complexity, rather than physical simplicity (part count), is associated with setup and takedown performance.

Our findings indicate that setup tasks (assembly and donning) consumed disproportionate amounts of time, imposed significant cognitive and physical demands on users, and present the primary barrier for deployment. The nearly four-fold spread in assembly times across devices suggests that device design characteristics may strongly influence deployment feasibility. This finding has important implications for industrial settings where shift changes, task rotations, and productivity demands can create tight time constraints and disincentivize systems with extended assembly times [63].

We think several exoskeleton specific design features also affected assembly times for each device (Table 9). For example, the Laevo and Skelex require extensive body measurements and sizing calculations before assembly can even begin. This need for measurement can create barriers for users who may lack appropriate measuring tools or have difficulties taking accurate self-measurements, or when measurements change because the user is wearing different clothing. Similarly, the need for specific tools, such as the Laevo’s requirement for hex keys and specific torque settings, can create barriers when tools may not be readily available or users lack the technical knowledge to apply proper specifications. Furthermore, in assembly procedures that involve sequential dependencies, any error early in the process can propagate, requiring partial or complete disassembly before restarting. This is the case for the Skelex, for which the assembly process progresses from belt to flexframe to Minax. Some devices also require fine motor control and technical understanding during assembly, such as the Skelex’s need for 180-degree rotations, specific window alignments, and precise force adjustments. Similarly, donning the Chairless Chair requires the user to put themselves in complex configurations, and the Laevo and Skelex require additional personnel to assist the user with donning. To don the Laevo, the vest frames, padding, connectors, and torso structures must be simultaneously coordinated simultaneously, forcing the user to manage multiple components while also positioning their body correctly. Finally, exoskeletons like the Skelex demand physical strength for handling heavy components or tight-fitting mechanisms. As with the assembly requirements, these barriers to donning could all lead to longer completion times and high failure rates for exoskeleton setup. These demands may create additional challenges for users facing time pressure.

**Table 9:** Design complexity comparison across exoskeletons.

Exoskeleton	HTA Steps (Total)	Usability Problems	Part Count	Setup Time (min)	Setup Success	Key Barrier
Ironhand	55	34	20	10.1	100%	Minimal
Chairless Chair	77	51	22	16.1	96.6%	Moderate
Skelex	73	66	5	20.8	72.4%	Precision demands
Laevo	136	41	22	27.4	50.0%	Tools, measurements

Note. Setup Time = Assembly + Donning. Setup Success = percentage completing both assembly and donning. Skelex demonstrates that low part count (5 parts) does not guarantee ease of use when procedural complexity and usability barriers are high.

Our findings also suggest that the number of task steps is a better predictor of success than the number of parts. The strong relationship between number of steps and assembly task completion times (R^2^ = 0.995) and failure rates (R^2^ = 0.968) underscores that the number of discrete task steps is associated with deployment success. The number of task steps and usability problems shaped by the design characteristics influences the cognitive load on the user, both intrinsic and extraneous. Intrinsic cognitive load refers to the mental effort inherent to the task itself, while extraneous cognitive load refers to additional mental effort imposed by poor design features such as unclear feedback, complex interfaces, or cumbersome processes that do not directly contribute to task performance. The Ironhand imposes a low intrinsic cognitive load because of its simple steps and a low extraneous load because of its clear feedback to the user, resulting in lower assembly times and higher completion rates. In contrast, the Laevo imposes a high intrinsic load due to its extended assembly sequence along with a high extraneous load due to its tool use requirements. As a result, task completion times were often higher for the Laevo while completion rates were lower. The Skelex has a more moderate intrinsic load but a high extraneous load because of the precision required for system setup tasks. Finally, the Chairless Chair has a similarly moderate intrinsic load but a low extraneous load, resulting in lower task completion times and higher completion rates. Each additional step in the assembly sequence adds to intrinsic cognitive load, demanding that users retain more sequential information while coordinating motor actions to align parts, secure fasteners, or adjust fit. Even simple actions can become performance bottlenecks when embedded within long, non-intuitive sequences. These insights extend cognitive load theory [64] to physical assembly tasks, where increased processing of discrete elements can heighten cognitive demand and reduce efficiency. Such cognitive strain could be exacerbated in dynamic, real-world environments where distractions, interruptions, or physical constraints are frequent.

Our findings on the demands introduced by number of task steps align with previous human factors studies performance in complex equipment assembly, which found that performance declines as attentional demands due to high step counts and the need for precise action sequencing increases [65]. As task complexity increases, users devote more cognitive resources to visual search and attentional control, slowing task execution and increasing task error rates. In our case, the number of task steps not only prolonged assembly but also increased the likelihood of cascading errors, as users had to repeatedly shift attention between locating components, orienting them correctly, and remembering the next step. These insights reinforce the need for exoskeletons with fewer assembly steps, intuitive sequences, and built-in attentional cues to lower cognitive demands and improve setup speed and success.

Our results also challenge assumptions that physical simplicity (fewer parts) guarantees ease of use. We found that the number of components in an exoskeleton (part count) was not significantly associated with task completion times or rates for any of the four setup and takedown tasks we considered. For example, the Skelex has only 5 parts but had longer assembly times and lower assembly success rates than the Ironhand, which has 20 parts but half as many assembly steps. The disconnect between part count and number of task steps suggests that the cognitive architecture of assembly procedures—that is, how parts and components relate to each other, the clarity of the assembly sequences, and the intuitiveness of the connections among parts—may have a more significant effect on the user than the number of physical parts.

Our findings also align with Norman’s conceptual model of design, which emphasizes that perceived simplicity depends more on cognitive factors (i.e., the number of task steps and the corresponding action sequence) than on physical attributes (i.e., part count) [66]. For example, the Ironhand’s modular design—with magnetic attachments and clear visual indicators—exemplifies how thoughtful design can make even multi-component systems accessible to users. This approach is advocated by design frameworks like the axiomatic design theory [67]. Conversely, the Skelex’s requirement for 180-degree rotations and force adjustments demonstrates how even simple devices can impose complex demands on the user, supporting previous observations that mechanical simplicity does not guarantee user-friendly interaction [68].

Our study highlights that the factors encompassing task complexity – number of steps, usability problems and part count appear to have differing impacts both within and across setup and takedown tasks. While assembly times were strongly associated with number of steps (R^2^ = 0.995), donning times were more strongly associated with usability problems (R^2^ = 0.854), suggesting that assembly tasks primarily challenge cognitive processing while donning tasks challenge motor execution. These exploratory patterns suggest that assembly procedures may benefit from reducing or simplifying the number and sequence of assembly steps, consistent with research on procedural learning. In contrast, donning procedures require attention to physical interactions, body positioning, and component adjustability, reflecting principles from usability and ergonomic design literature. This distinction has not previously been documented in exoskeleton research, which has typically treated setup as a single task [5,37]. These differential challenges across the two setup task phases also suggest a need for task-specific training approaches not currently considered in current exoskeleton certification programs [35].

Compared to the setup tasks, the relative ease of takedown tasks (doffing and disassembly) likely reflects both inherent task differences and the learning effect and reduced cognitive load once users have successfully navigated setup procedures. Because participants completed setup immediately before takedown with each device, faster takedown times may partly reflect recall of component locations and connection points rather than inherent simplicity of takedown procedures. However, the moderate to large effect sizes for disassembly times between devices (Cohen’s d = 0.77 to 1.75) indicate that even these simpler tasks present design challenges that could impact end-of-shift workflows and device storage procedures.

In summary, devices requiring extensive measurements, specialized tools, greater number of task steps, sequential dependencies and usability problems tend to require longer setup times and are associated with high failure rates, and thus can be a barrier to deployment, while devices with intuitive connections and feedback mechanisms can enable rapid, reliable deployment. These findings support design principles such as minimizing measurement dependencies, eliminating tool requirements, and designing in error-tolerant and constraint mapped connectors—principles that may directly inform next-generation exoskeleton development.

In the following sections, we provide design and deployment recommendations for wearable devices such as exoskeletons. These recommendations extend the design guidelines proposed by [35] and the ASTM standards for exoskeleton evaluation.

### 4.1. Design, deployment and theoretical implications

#### 4.1.1. Recommendations for exoskeleton design.

**Minimize measurement dependencies.** Devices that require extensive anthropometric measurements (such as the Laevo) introduce barriers before assembly can even begin. However, several design strategies can overcome these obstacles. One such strategy involves modifying designs to enable self-sizing and automatic adjustments. For example, fixed-length straps or other components that need a tape measure for fitting can be replaced with micro-adjustable ratchet or dial systems so that user can quickly fine-tune the fit without tools. Tensioned elastic adjustment points or spring mechanisms can also be used to fit a range of body sizes without manual measurement. Similarly, arm and leg support that slide and lock into place (e.g., using sliding or telescoping elements and predefined detents) enable customized fitting without requiring precise measurements.

Designers can further reduce the setup burden by developing systems for which the default device settings accommodate a majority of the workforce without the need for custom adjustments (using NHANES or NASA anthropometric databases). Visual and tactile fit indicators—including color-coded green zone guides that indicate optimal fit ranges, audible or tactile clicks that confirm correct positioning, and pre-labeled adjustment markings (e.g., short, medium, tall)—can further simplify fitting. Where possible, devices could incorporate single-point adjustment systems that proportionally adjusts multiple straps. In addition to these design considerations, the time demands for setup tasks could be further reduced using systems that store worker settings for reuse and digital fit assistants (e.g., QR codes or mobile apps) that walk workers through a fit check with visual prompts.

**Eliminate tool requirements.** The need for hex keys, torque specifications, or other tools adds to the task time and task complexity. To minimize these setup and takedown burdens, devices could integrate tool-free fastening and tensioning systems such as thumb-screws, quick-release pins, cam-locks, lever clamps, and spring-loaded latches and detents. Similarly, self-limiting tightening mechanisms such as pre-set torque clips and elastic or ratchet straps could help with quick setup and release. Important joints can be built with modular, drop-in components that incorporate keyed connections (i.e., shaped joints that allow only one orientation for insertion) or magnetic alignment aids that guide parts into the correct position before locking. Designers can also reduce the number of adjustable joints by linking multiple adjustments together (e.g., where a single strap simultaneously alters shoulder, chest, and waist tension) and use fixed-range geometry principles to design components that fit standard anthropometric ranges.

**Reduce sequential dependencies.** Assembly procedures that depend on accurate, sequential actions can risk cascading errors that amplify the impact of minor mistakes. To minimize this risk and improve setup times, devices should be developed following error-tolerant design principles. For example, devices can be designed as modular, independent subassemblies that can be connected in any order, allowing errors to be corrected without complete disassembly. Such designs would also allow for multiple start points depending on user preferences. Error-tolerant connectors, including reversible fasteners, self-aligning shape joints, and reconfigurable or swappable components with symmetrical parts and universal attachments, could further prevent misalignments. In addition, exoskeletons could include built-in feedback and guidance with visual or tactile progressive locking indicators and digital verification steps (e.g., with QR codes or NFC tags) to guide users and confirm step completion. Single-action adjustment systems and redundant error recovery paths can also significantly minimize setup time and effort.

**Prioritize intuitive connections.** The Ironhand exemplifies how haptic and auditory feedback (e.g., magnetic attachments and click mechanisms) can guide users through the assembly process without requiring extensive instructions or training. Visual reinforcement, such as alignment markers or color-changing materials or coatings, could also indicate when a step is complete. Following affordance-based shaping principles for connection points and incorporating follow-the-path assembly cues (with channels, grooves, and rails to physically guide parts into the correct position and orientation) could also minimize setup time and effort.

**Consider multi-user requirements.** Devices that require assistance for donning or doffing (like the Laevo) impose additional labor costs that may be impractical in settings where workers operate independently. Solo exoskeleton operation could be supported by incorporating one-handed adjustment mechanisms and pursuing gravity-assisted donning designs with open, rigid frames that remain upright for the user to step into and secure the device without assistance. Self-aligning joints with indexed pivot points and asymmetrical shapes, auto-latching mechanisms that lock into place when parts are in proximity without requiring precise positioning, and guided positioning fixtures and counterbalance supports during donning can further minimize the time and effort required for setup and takedown.

#### 4.1.2. Recommendations for industry deployment.

Our results suggest that exoskeleton designs should prioritize procedural simplification using modular product architectures for setup and takedown alongside traditional considerations such as biomechanical effectiveness [69–71]. In our study, participant success rates with the Ironhand (100% task completion rate with only 25 assembly steps) demonstrates advanced functionality does not inherently require task complexity. In contrast, the high setup failure rates for the Laevo, which required 65 steps for precise alignment and tensioning, may not only delay deployment in practical settings but also risk complete abandonment of the technology, as workers facing repeated setup failures may develop negative perceptions that persist beyond their initial training. Indeed, technology acceptance research has shown that initial user experiences strongly predict long-term adoption [72–74].

Setup time can also have important implications in industrial scenarios where a single exoskeleton may be shared across multiple workers and shifts, a deployment model that will likely be common given that exoskeleton costs range from $3,000 to $10,000 per unit. Unlike traditional ergonomic interventions that involve one-time modifications to tasks, equipment, or environments, exoskeletons may require repeated setup and takedown cycles that can compound time losses, thereby adding temporal efficiency to the cost-benefit analysis that has historically focused solely on biomechanical effectiveness. In industrial settings when exoskeletons may be used for intermittent but repeated tasks such as periodic lifting (e.g., warehousing), overhead work (e.g., construction), or any other setting requiring multiple donning/doffing events per shift (e.g., taking breaks, switching tasks, or following safety protocols), the ratio of setup time to productive usage time in a typical 8-hour shift can be a critical determinant of deployment feasibility. The design characteristics that facilitate or hinder rapid deployment and disassembly correspondingly affect the feasibility of integrating these devices into existing operational procedures.

Storage and maintenance considerations impose an additional demand on the setup and takedown task scenarios. Industrial environments often have limited storage, which necessitates efficient exoskeleton disassembly for compact storage between uses. The ability to quickly disassemble the devices for cleaning, maintenance, and storage is particularly important in healthcare settings with strict hygiene protocols and construction environments where equipment needs to be secured and transported between job sites.

Building on these insights, we introduce *deployment ergonomics* as a complementary perspective to understand how the setup and takedown phases shape real-world deployment and sustained use of wearable technologies such as exoskeletons. While traditional ergonomics frameworks focus on optimizing performance, safety and comfort during device operation, deployment ergonomics extends these principles to setup and takedown phases, emphasizing the ease, speed, and reliability with which users can prepare and disengage technologies for use. This perspective emphasizes deployability – the cognitive effort and efficiency required for device initialization and disengagement as a measurable and designable construct that can influence user acceptance and adoption. In doing so, deployment ergonomics aligns with and extends existing ergonomics approaches advocating a full lifecycle approach to worker-device interaction for successful adoption. Our findings indicate that design factors such as the number of steps, part count and usability problems, along with task outcomes such as time, success rates and errors can serve as critical metrics for assessing deployability. Viewing setup and takedown tasks through this lens underscores that human centered design should not stop at operational elements but also consider design for deployment.

#### 4.1.3. Theoretical and methodological implications.

Our results extend ergonomics literature on exoskeleton adoption by highlighting the critical role of device setup and takedown procedures in technology acceptance. We propose that technology adoption barriers exist in two distinct phases: initialization barriers (setup and takedown) and operational barriers (during use). Traditional technology acceptance models including TAM [72] and UTAUT [74] primarily address operational barriers such as perceived usefulness and ease of use during task execution. However, these models assume users have already successfully initialized the technology. By showing that the task complexity demands involved in setup and initialization can decisively shape adoption trajectories, our insights contribute to and broaden models of technology acceptance [74] suggesting that ease of setup and takedown should be considered as a distinct construct in wearable technology acceptance models, particularly for devices intended for repeated daily use. Operational barriers affect interaction during productive work, but users typically receive performance feedback, and can experience the technology’s benefits directly. But initialization barriers create a “deployment constraint” that prevents users from even reaching the operational phase. In particular, during setup and takedown, there is no performance feedback, and no direct benefits from the technology. In addition, if there is high cognitive load and increased time investment in the initialization phase, we think that will impact acceptance and eventual deployment. These insights extend prior work on exoskeleton acceptance [75] and the organizational factors influencing exoskeleton implementation [76].

### 4.2. Study limitations and future directions

Several limitations should be considered when interpreting our findings. First, regarding our statistical approach: our design-level regression analyses (N = 16 exoskeleton-task combinations) and task-specific analyses (N = 4 exoskeletons per task) had limited statistical power. We addressed this concern by conducting participant-level analyses using all 397 individual observations, which confirmed the key findings with more conservative effect size estimates (R² = 0.669 vs. 0.753 at the design level). Within-participant correlations (mean r = 0.850) provided additional evidence that the complexity-performance relationship holds within individuals, not merely between experimental conditions. Nevertheless, the task-specific findings (e.g., assembly R² = 0.995, donning usability R² = 0.854) should be interpreted as exploratory patterns requiring confirmation with larger samples of exoskeleton designs.

The ecological validity of our findings is limited in several respects. Our study was conducted in a controlled laboratory environment with dedicated time and space for setup and takedown tasks. Real-world industrial settings may impose additional constraints, such as limited space, time pressure, or other environmental factors, that could significantly influence task complexity and exacerbate the challenges we observed [2,63]. Indeed, field studies have shown that laboratory findings may underestimate real-world challenges [42,77]. Additionally, participants were engineering students rather than industrial workers. While this ensured consistent technical background, engineering students may be more comfortable with technical assembly and problem-solving than typical production workers, potentially leading to faster completion times and higher success rates than would be observed in industrial populations. The two-hour testing session also does not capture the repetitive deployment cycles that would occur over days or weeks of industrial use. Our findings should therefore be interpreted as establishing associations under controlled conditions, with field validation studies needed to confirm generalizability.

The faster completion of takedown tasks compared to setup tasks likely reflects both inherent task differences and a memory/familiarity confound. Because participants completed setup immediately before takedown with each device, faster takedown times may partly reflect recall of component locations and connection points rather than inherent simplicity of takedown procedures. Although our order effects analysis showed no significant cross-device learning (p = .258) and the HTA-time relationship remained significant after controlling for presentation order (partial r = 0.814, p < .001), designs that counterbalance setup and takedown order would be needed to fully isolate inherent task difficulty from learning effects.

Another limitation in our study is that our four exoskeletons span different body regions (hand, shoulder, trunk, lower limb), introducing confounds that limit cross-device generalization. Devices supporting different body regions may have inherently different fitting requirements: a trunk exoskeleton may require more adjustment points than a hand exoskeleton due to biomechanical necessity rather than suboptimal design. The high step count for the Laevo (136 total steps) may reflect the genuine complexity of properly fitting a trunk support device rather than design inefficiency. Our finding that procedural step count predicts completion time may therefore partly reflect these inherent differences rather than isolable design characteristics. Comparing devices within a single category would have allowed more direct isolation of design complexity effects while controlling for anatomical and task-inherent differences associated with different body regions. However, our approach offers complementary value: by demonstrating that procedural complexity (HTA steps) strongly predicted completion times across diverse exoskeleton types, our findings suggest that the relationship between design complexity and deployment burden generalizes across the broader exoskeleton landscape rather than being specific to one device category. Future studies should examine whether these relationships hold within specific exoskeleton categories using multiple devices of the same type.

Our complexity measures have construct validity limitations. HTA step counts depend on decomposition granularity, and while our consensus-based methodology ensured consistency across devices, different analysts might produce different absolute counts. Usability problems were counted without severity weighting; a cosmetic interface issue received equal weight to a critical assembly barrier. Future research should explore severity-weighted usability indices. Part count proved uninformative as a predictor, likely because simple counts do not capture functional coupling between parts, modularity of subassemblies, or adjustment complexity. More sophisticated design metrics from the design-for-assembly literature may better predict performance.

Our binary success definition (completed within 30 minutes vs. not completed) conflates true inability, cautious behavior, instruction ambiguity, and time constraints. The 30-minute cutoff was practically motivated but may have truncated some participants who would eventually have succeeded. Future studies could employ finer-grained outcome measures such as error counts, assistance requests, or time-to-completion distributions.

To address these limitations, future studies should investigate setup procedures under varying time pressures to better approximate real-world deployment scenarios [61,78]. In addition, longitudinal studies would help reveal how task complexity influences setup times and success rates with extended use, thereby providing insights into longer-term learning curves and the persistence of usability problems [79]. Studies that investigate the relationship between task complexity in setup and takedown and long-term adoption rates, as well as the viability of shared-use deployment models in industries with multiple shifts and rapid changeovers, could help quantify the economic implications of design decisions and identify design characteristics that facilitate or hinder rapid assembly and disassembly. Such studies will be particularly important given the unique economic challenges of exoskeleton implementation relative to other workplace interventions.

Finally, future research should consider different work cultures and a greater diversity of industrial sectors including manufacturing, healthcare, and construction. The development of standardized metrics for setup and takedown would also facilitate comparative studies across devices and guide design optimization for practical deployment.

## 5. Conclusions

In conclusion, our results show that the promise of exoskeletons for reducing workplace injuries may be undermined by its setup and takedown procedures. The substantial time requirements, high task failure rates, and elevated task complexity of the setup tasks associated with some devices suggest that current exoskeleton designs may be incompatible with the operational realities of industrial settings. This study generates new knowledge and implications for design practice and ergonomics research that setup and takedown procedures are not mere peripheral considerations, but present critical barriers to deployment. Our findings demonstrate that reducing the number of task steps, rather than minimizing part counts or adding technological sophistication, should be a central design priority for improving deployability. Setup times differed by nearly four-fold among exoskeletons, and completion rates varied by almost 50%, highlighting that these challenges come from design choices rather than inherent technological limitations. As the exoskeleton industry matures, competitive advantages may accrue not to devices with the most advanced assistive functions, but to those that workers can deploy efficiently within real-world temporal, cognitive and economic constraints. Ultimately, widespread adoption may depend less on technological sophistication and more on deployment ergonomics – the design of exoskeletons that are not only effective, but also deployable across diverse industrial environments with intuitive, time-efficient and easy setup and takedown tasks that ensure exoskeletons fulfill their promise of reducing injuries, improving safety and productivity.

## Supporting information

S1 File Hierarchical Task Analysis. (PDF)
