# Supplementary material for: Task complexity in exoskeleton setup and takedown: Procedural steps and usability problems as predictors of deployment performance
Source: PLoS One. 2026 Apr 28;21(4):e0348001. doi: 10.1371/journal.pone.0348001 (PMC13123993; doi:10.1371/journal.pone.0348001)
Supplement: S1 File — (PDF) [file pone.0348001.s001.pdf]

HTA Table of Contents

IRONHAND 2

CHAIRLESS CHAIR 8

LAEVO 11

SKELEX 21

IRONHAND

0. Assemble, don, doff, and disassemble Ironhand

Plan 0. Do 1, 2, 3, 4 in order

1. Assemble Ironhand

Plan 1. Do 1.1., 1.2., 1.3., in order, if using backpack do 1.4. If using hip carry do 1.5. If wearing two gloves (left and right) do all.

1.1. Check battery status and charge battery

Plan 1.1. Do 1.1.1., 1.1.2, 1.1.3 in order; if 1.1.3.1. indicates solid ORANGE, do 1.1.3.2. and 1.1.3.3. If 1.1.3.1. indicates solid GREEN, do 1.1.3.3. If 1.1.3.1. indicates other colors, consult the manual and/or the manufacturer for further actions.

1.1.1. Take battery out of the Ironhand case

1.1.2. Insert battery into charger

1.1.3. Plug charger into wall outlet

1.1.3.1. Check battery indicator light color

1.1.3.2. Wait for battery to charge

1.1.3.3. Remove battery from charger

1.2. Insert charged battery into battery power pack

1.2.1. Locate the grooved surface on both sides of the powerpack

1.2.2. Use your thumb and forefinger to grasp the grooved surface

1.2.3. Lift up the battery compartment cover

1.2.4. Visually identify and locate the metal contact side on the battery

1.2.5. Orient battery to put into compartment such that metal contacts are at the left lower corner facing away from the cover

1.2.6. Slide battery into battery compartment

1.2.7. Press down on cover to close battery compartment

1.3. Connect your size glove to power pack

Plan 1.3. Do 1.3.1 first, then do 1.3.2. If 1.3.2. is true, then 1.3.3., else 1.3.5. If 1.3.3. is your glove size, exit 1.3. If 1.3.3. is not your glove size, do 1.3.4., then do 1.3.5., 1.3.6, 1.3.7., 1.3.8. in order.

1.3.1. Determine your glove size (from small, medium, large, extra large) for the appropriate hand (right and/or left).

1.3.2. Check if a glove connector is connected with the power pack.

1.3.3. Determine the size of the glove attached to the glove connector.

1.3.4. Remove the power pack from the glove connector

1.3.4.1. Place one hand on the bottom of the glove connector

1.3.4.2. Grip the power pack using the grooves provided on the top end.

1.3.4.3. Pull the power pack with force to detach from glove connector.

1.3.5. Locate the glove connector base with desired glove size.

1.3.6. Lay the glove connector base on a flat surface like a table with the grooves facing up.

1.3.7. Lower and place the battery pack into the glove connector base, orienting the cable in the battery pack in the same side as the cable in the glove connector base.

1.3.8. Join and click the battery pack with the glove connector base

1.4. Prepare the backpack

1.4.1. Remove backpack from case.

1.4.2. Place backpack on flat surface like a table with shoulder slings facing downward

1.4.3. Open zipper to access battery pouch compartment

1.4.4. Slide in battery pack-glove connector assembly with battery cover facing up and oriented to the bottom with wires oriented at the top of the backpack

1.4.5. Close the zipper

1.4.6. Press down the cord connecting the power pack base with the glove into the cord clips on the backpack to secure it.

1.4.7. Slide down the remote control into the remote control holder in the backpack sling to secure it so it does not dangle.

1.5. Prepare the hip carry

1.5.1. Remove hipcarry from case.

1.5.2. Place hipcarry on flat surface like a table with hip belts facing away from you.

1.5.3. Open the Velcro to access the battery pouch compartment.

1.5.4. Open the zipper to the battery pouch compartment.

1.5.5. Slide in battery pack-glove connector assembly with battery cover facing up with wires oriented at the top of the hipcarry.

1.5.6. Close the zipper.

1.5.7. Route the cord connecting the power pack base to the glove and the wire connecting the remote control to the battery pack through either the right side slit (for right hand glove) or the left side slit (for left hand glove) in the hip carry.

1.5.8. Press down the cord connecting the power pack base with the glove into the cord clips on the hipcarry to secure it.

1.5.9. Loop the remote control through the remote control loop in the hipcarry sling to secure it so it does not dangle.

1.5.10. Buckle the 3 shoulder harness buckles on the top side of the hip carry pack.

2. Don the Ironhand

Plan 2. If using only a backpack, do 2.1. If using only a hipcarry, do 2.2. If using both, do 2.2. and then 2.1. in order.

2.1. Don the Ironhand using the backpack.

2.1.1. Wash hands and use hand sanitizer.

2.1.2. Sling the Ironhand backpack over shoulders like one would a backpack.

2.1.3. Adjust the slings on the shoulders and bring the sternum straps together.

2.1.4 Click the top and the bottom sternum straps together

2.1.5. Adjust the length of the straps so the backpack slings sit tight on the shoulder.

2.1.6. Put on the glove.

2.1.7. Adjust the tightness of the glove on the wrist by pulling the strap on the glove.

2.1.8. Slide the longer arm strap over the glove cord and put the arm strap in the upper arm.

2.1.9. Tighten the arm strap in the upper arm by pulling on the strap and attaching it to the Velcro.

2.1.10. Slide the shorter arm strap over the glove cord and put the arm strap in the forearm.

2.1.11. Tighten the arm strap in the forearm by pulling on the strap and attaching it to the Velcro.

2.1.12. Locate the outer glove size from the box.

2.1.13. Put on the outer glove on top of the Ironhand glove.

2.1.14. Adjust wire length as needed by sliding the wires inside the arm straps on the shoulder.

2.2. Don the Ironhand using the Hipcarry

2.2.1. Wash hands and use hand sanitizer.

2.2.2. Take assembled hip carry belt and buckle the belt around the hips.

2.2.3. Tighten the belt around the hip.

2.2.4. Put the shoulder harness around the shoulders so that the hip carry is in the back near the hip.

2.2.5. Slide the longer arm strap over the glove cord and put the arm strap in the upper arm.

2.2.6. Tighten the arm strap in the upper arm by pulling on the strap and attaching it to the Velcro.

2.2.7. Slide the shorter arm strap over the glove cord and put the arm strap in the forearm.

2.2.8. Tighten the arm strap in the forearm by pulling on the strap and attaching it to the Velcro.

2.2.9. Clip in and secure the wire that connects the hip carry to the glove into the arm strap clips in the upper arm and forearm arm straps.

2.2.10. Put on the glove.

2.2.11. Adjust the tightness of the glove on the wrist by pulling the straps on the glove.

2.2.12. Locate your size outer glove from the box.

2.2.13. Put on the outer glove on top of the Ironhand glove.

2.2.14. Adjust wire length as needed by sliding the wires inside the arm straps on the hip carry.

3. Doff the Ironhand

Plan 3. If using only a backpack, do 3.1. If using only a hipcarry, do 3.2. If using both, do 3.1. and 3.2. in order.

3.1. Doff the Ironhand with the backpack

3.1.1. Remove the outer glove.

3.1.2. Detach the Velcro of the forearm arm strap and slide it down to remove.

3.1.3. Detach the Velcro of the upper arm strap and slide it down to remove.

3.1.4. Unclip cord that is clipped in the shoulder part of the sling and make the cord connecting the battery pack with the glove loose.

3.1.5. Loosen the strap on the ironhand glove

3.1.6. Place opposite hand's thumb beneath glove from the side of the inner wrist to loosen it. Then, pull lightly on each finger to take it off.

3.1.7. Unbuckle the sternum top and bottom straps.

3.1.8. Sling out the backpack from the shoulders to place device on a surface like a table.

3.2. Doff the Ironhand with the hipcarry

3.2.1. Remove the outer glove.

3.2.2. Detach the Velcro of the forearm arm strap and slide it down to remove.

3.2.3. Detach the Velcro of the upper arm strap and slide it down to remove.

3.2.4. Loosen the strap on the ironhand glove

3.2.5. Place opposites hand's thumb beneath glove from the side of the inner wrist to loosen it. Then, pull lightly on each finger to take it off.

3.2.6. Remove the shoulder harness from the shoulders.

3.2.7. Unbuckle the hip belt.

3.2.8. Remove the hip belt Velcro.

3.2.9. Place the hip carry and the device on a surface like a table.

4. Disassemble the Ironhand

Plan 4. If using only a backpack, do 4.1. If using only a hipcarry, do 4.2. If using both, do 4.1. and 4.2. in no specific order.

4.1. Disassemble the Ironhand with the backpack

4.1.1. Unclip the remote control from the clip holding it in place.

4.1.2. Open the zipper in the backpack that contains the powerpack.

4.1.3. Remove the powerpack from the backpack.

4.1.4. Open the battery compartment that keeps the battery in the powerpack.

4.1.5. Once the battery pops out, slide and remove the battery from the battery compartment.

4.1.6. Close the battery compartment cover.

4.1.7. Remove the power pack from the glove connector

4.1.7.1. Place one hand on the bottom of the glove connector.

4.1.7.2. Put the other hand on top of the power pack, such that you have a good grip at it.

4.1.7.3. Pull the power pack with some force to detach from glove connector.

4.1.8. Store all parts in case.

4.2. Disassemble the Ironhand with the hip carry

4.2.1. Open the zipper in the hip carry that contains the powerpack.

4.2.2. Remove the powerpack from the hip carry.

4.2.3. Open the battery compartment that keeps the battery in the powerpack.

4.2.4. Once the battery pops out, slide and remove the battery from the battery compartment.

4.2.5. Close the battery compartment cover.

4.2.6. Remove the power pack from the glove connector

4.2.6.1. Place one hand on the bottom of the glove connector.

4.2.6.2. Put the other hand on top of the power pack, such that you have a good grip on it.

4.2.6.3. Pull the power pack with some force to detach from glove connector.

4.2.7. Store all parts in case.

CHAIRLESS CHAIR

0. Assemble, don, doff, and disassemble Chairless Chair

Plan 0. Do 1, 2, 3, 4 in order.

1. Assemble Chairless Chair

Plan 1. If using both the waist and shoulder belts, do 1.1 through 1.9 and then 1.13. through 1.20 and skip 1.10 through 1.12. If using only the waist belt, do 1.1 through 1.7, skip 1.8 through 1.12., and do 1.13 through 1.20 in order. If using only the shoulder belt, do 1.1 through 1.5., skip 1.6 through 1.9, and do 1.10 through 1.20 in order. Do 1.20 only if tightening is needed.

1.1. Place the frame of the chairless chair facing the seat pads downwards in a flat surface.

1.1.1. Make sure that the orange lever on the leg frame is facing outwards.

1.2. Slide any slider into the bottom end of the lower left leg frame, having it point downward and the magnet facing inwards.

1.3. Slide the rubber feet into the lower left leg frame after the slider and push the rubber feet firmly.

1.4. Slide the other slider into the bottom end of the lower right leg frame, having it point downward and the magnet facing inwards.

1.5. Slide the rubber feet into the lower right leg frame after the slider and push the rubber feet firmly.

1.6. Locate and grab the waist belt.

1.7. Align and buckle the waist belt buckles into the corresponding buckles in the seat pads so that the tension adjusters are on the outside.

1.7.1. If waist measurement is between 107cm - 137 cm, add the waist extender.

1.7.1.1. Attach the extender to one side of the waist belt by joining the velcros on the waist belt and the extender.

1.8. Attach the buckle ends of the shoulder belt into the corresponding buckles in the waist belt.

1.9. Hook the hangers on the shoulder belt into the back side of the waist belt beneath the tension adjusters where the hooks are located, one on the right side and the other on the left side.

1.9.1. Make sure the hangers are hooked in completely, a clicking sound must be heard.

1.10. Attach the buckle ends of the shoulder belt into the corresponding buckles in the shoulder vest-seat connector pad.

1.11. Hook the hangers on the shoulder belt into the back side of the vest-seat connector pad where the hooks are located, one on the right side and the other on the left side.

1.12. Attach the buckle ends of the vest-seat connector pad into the corresponding buckles in the seat.

1.13. Attach the left leg straps to the left seat.

1.14. Attach the right leg straps to the right seat.

1.15. Attach the left side seat pads to the seats using the Velcro backing in the seat pads.

1.16. Attach the right side seat pads to the seats using the Velcro backing in the seat pads.

1.17. Use a measuring tape to measure individual's height and record it on paper.

1.17.1. Choose XS if height size is between 150-159cm / 4ft 11'' – 5ft 2''.

1.17.2. Choose S if height size is between 160-169cm / 5ft 3'' – 5ft 6''.

1.17.3. Choose M if height size is between 170-184cm / 5ft 7'' – 6ft 0''.

1.17.4. Choose L if height size is between 185-200cm / 6ft 1'' – 6ft 7''.

1.18. Press down on the orange size adjustment button on the lower frame and slide the lower frame to align the frame's square slot into the size based on height.

1.19. Press down on the orange size adjustment button on the upper frame and slide the upper frame to align the frame's square slot into the size based on height.

1.20. Adjust the seat pad belt lengths to the same length by pulling up or down the belt with the buckles as appropriate.

2. Don the chairless chair

Plan 2: If using both the waist and shoulder belts, do 2.1 through 2.21. If using only the waist belt, do 2.1 through 2.12, skip 2.13 through 2.16, and do 2.17 through 2.21. If using only the shoulder belt do 2.1 through 2.9, skip 2.10 through 2.12 and do 2.13 through 2.21 in order.

2.1. Locate the right and the left shoe clickers.

2.2. For the right shoe clicker, align the clicker port to be on the outward side of the right shoe and have the orange tab facing upwards.

2.3. Fasten the buckle of the right shoe clicker over the top of the right shoe such that one strap goes over the back of the shoe and the other on top so that the black flat surface is on the inside of the shoe facing inward.

2.4. Adjust the tension of the right shoe clicker with the front and back shoe straps.

2.5. Pull straps tight and tuck away any loose ends.

2.6. For the left shoe clicker, align the clicker port to be on the outward side of the left shoe and have the orange tab facing upwards.

2.7. Fasten the buckle of the left shoe clicker over the top of the left shoe such that one strap goes over the back of the shoe and the other on top so that the black flat surface is on the inside of the shoe facing inward.

2.8. Adjust the tension of the left shoe clicker with the front and back shoe straps.

2.9. Pull straps tight and tuck away any loose ends.

2.10. Use both hands to hold the chairless chair by the waist belt vertically in front of you.

2.11. While still holding the chairless chair by the waist belt vertically, move the chairless chair to the back side and wear the waist belt just above the hips and beneath the waist.

2.12. Pull and tighten the waist belt further to ensure that it is secure above the hips.

2.13. Use both hands to hold the chairless chair by the shoulder belt vertically in front of you.

2.14. While still holding the chairless chair by the shoulder belt vertically, move the chairless chair to the back side.

2.15. Slide the shoulder belt slings on the left and right sides into the left and right shoulders.

2.16. Tighten the shoulder straps on left and right sides by pulling the strap ends on the left and right sides.

2.17. Pull the left and right tension adjusters on the waist belt forward to the desired fit and attach the ends of the adjusters to the belt.

2.19. Stand in a normal posture and hold the right leg frame and guide the right slider magnet into the right clicker port.

2.20. Stand in a normal posture and hold the left leg frame and guide the left slider magnet into the left clicker port.

2.21. Close the right leg straps with the shorter part of the strap on top.

> 2.21.1. If a leg strap extension is needed, attach it by joining the Velcro of the extension to one side of the leg strap.

2.22. Close the left leg straps with the shorter part on top.

> 2.22.1. If a leg strap extension is needed, attach it by joining the Velcro of the strap extension to one side of the leg strap.

3. Doff the chairless chair

Plan 3: If using both waist and shoulder belts, do 3.1 to 3.10 in order. If using only the waist belt, do 3.1. to 3.6 in order, skip 3.7, and do 3.8 to 3.10 in order. If using only the shoulder belt, do 3.1. to 3.4. in order, skip 3.5 and 3.6 and then do 3.7 to 3.10 in order.

3.1. Pull the orange tab on the right-side clicker and lift your right heel and slightly turn it outward to completely detach the slider from the clicker on the right side.

3.2. Pull the orange tab on the left side clicker and lift your left heel and slightly turn it outward to completely detach the slider from the clicker on the left side.

3.3. Open the right leg straps.

3.4. Open the left leg straps.

3.5. Loosen the waist belt tension adjusters.

3.6. Open the waist belt.

3.7. Remove the shoulder belt slings from the shoulders.

3.8. Remove the chairless chair from the body.

3.9. Take off shoe clicker on the right and left side.

4. Disassemble the chairless chair

Plan 4. If using both the waist and shoulder belt do 4.1 through 4.12 in order and skip 4.7. If using only the waist belt skip 4.5. through 4.7. If using only the shoulder belt, skip 4.8.

4.1. Remove the right and left side seat pads from the seat.

4.3. Remove the right and left leg straps from the seat.

4.5. Remove the shoulder belt buckles from the waist buckles.

4.6. Unhook the shoulder belt from waist belt.

4.7. Remove the shoulder vest-seat pad connector from the seat pads.

4.8. Remove the waist belt from the seat pads.

4.9. Remove the right and left rubber feet from the lower leg frame.

4.10. Remove the right and left slider from the lower leg frame.

LAEVO

0. Assemble, don, doff, and disassemble Laevo

Plan 0: Do 1, 2, 3, 4 in order.

1. Assemble Laevo

Plan 1: If you do not know desired sizes do 1.1. If sizes are already known for the front vest, vest frame, torso structures, actuator springs, and leg pads then skip to 1.2. Then do 1.3. If vest frame padding is already installed skip 1.3. Then do 1.4. If the correct size for front vest is

already installed skip step 1.4. Then do 1.5. If the back connector is already attached to the lower bar skip 1.5. Then do 1.6., 1.7. and 1.8. If the correct actuator spring is already in the smart joint skip 1.8. Then do 1.9., 1.10 is optional.

1.1. Determine the necessary sizes needed by obtaining the correct measurements and writing them in the sizing form 1.1

1.1.1. Find the following bone locations to facilitate the process: tip of the shoulder (acromion), bottom of chest bone (sternum), hip joint (trochanter major), and kneecap (patella).

1.1.2. Measure the width of the torso on the height of the bottom of the chest bone in a horizontal, straight line. Keep hands flat and parallel on the sides of the chest and measure the distance between hands.

1.1.2.1 Write down the distance in cm.

1.1.3. Measure the width of the hip on the height of the hip joint in a horizontal, straight line. Keep hands flat and parallel on the sides of the chest and measure the distance between hands.

1.1.3.1 Write down the distance in cm.

1.1.4. Measure the distance from the tip of the shoulder to the hip joint in a straight line. Hold the ends of the measuring tape against the bone locations.

1.1.4.1 Write down the distance in cm.

1.1.5. Measure the total body length from the floor to the top of the head.

1.1.5.1 Write down the distance in cm.

1.1.6. Weigh yourself on a scale in kilograms (kg).

1.1.6.1 Write down the weight in kg.

1.1.7. Measure the distance from the hip joint to the top edge of the kneecap while letting the measuring tape rest and curve on the leg.

1.1.7.1 Write down the distance in cm.

1.1.8. Use the sizing form 1.1 to find the appropriate sizes for the front vest, vest frame, torso structures, actuator springs, and leg pads, using the sizes taken in previous steps.

1.2. Find and select the sizes needed for front vest, lower bar of vest frame, torso tubes, leg pads and actuator spring.

1.3. Installing the vest frame padding

1.3.1. Attach the vest frame padding to the top of the lower bar vest frame by opening the velcro in the padding and placing it over the lower bar vest frame.

1.3.2. Attach the vest frame padding to the bottom of the vest frame by opening the Velcro and placing it over the vest frame.

1.3.3. Pull and close tight velcros at all 4 edges of the vest frame to secure.

1.4. Installing the front vest

Plan 1.4. Do 1.4.1., 1.4.2., 1.4.3., 1.4.4. in order. If the straps are on the zipper and the exoskeleton is only used by one person then do 1.4.5., but this last step is optional.

1.4.1. To orient the front vest and place it appropriately, put the wide (lower) end of the front vest over the wide (lower) end of the vest frame.

1.4.2. Insert and pull the straps of a new front vest in the slots of the vest frame (4 straps).

1.4.3. Close the Velcro of the front vest straps.

1.4.4. Make sure that the Velcro has a minimum overlap of about 10 cm.

1.4.5. Cut the straps of the front vest at the blue lines.

1.5. Connecting the back connector to lower bar of vest frame

1.5.1. Connect the back connector to the vest frame by tilting it around the vest frame into the hole.

1.5.2. Slide the back connector from left to right until an audible click is heard and the con nector swivel is locked.

1.5.3. To ensure the back connector is secured, pull lightly on it in several directions.

1.6. Connecting the torso structures to back connector.

1.6.1. Push the lock button of the torso structure on the right part of the back connector, which is labelled ‘R’.

1.6.2. Put the right torso structure, labelled ‘R’, into the back connector. Slide it to the necessary length setting or use the middle setting.

1.6.3. Release the lock button. An audible click means the torso structure is locked.

1.6.4. Repeat steps 1.6.1. through 1.6.3. but now for the left side, labelled ‘L’.

1.6.5. Check that left and right torso structures are set to the same length.

1.6.6. Pull lightly on all torso structure ends to make sure all ends are locked.

1.7. Connecting the torso structures to the hip frame.

1.7.1. Put the left torso structure, labelled ‘L’, into the left smart joint, labelled ‘L’.

1.7.2. An audible click means the torso structure is locked.

1.7.3. Pull lightly on the torso structure to make sure that it is locked.

1.7.4. Repeat steps 1.7.1. through 1.7.3. but now for the right side, labelled ‘R’.

1.8. Putting in the actuator springs

Plan 1.8. If the incorrect actuator spring is already in the smart joint do 1.8.1 through 1.8.7. If there is no actuator spring in the smart joint skip step 1.8.2. only and do the rest.

1.8.1. Use an 8 mm hex key to remove the cap from the smart joint.

1.8.2. Remove the actuator spring from the smart joint.

1.8.3. Put the actuator spring in the smart joint.

1.8.4. Put the cap in the hole at the top of the smart joint.

1.8.5. Use the supplied 8 mm hex key to tighten until the cap head contacts the smart joint.

1.8.6. Check that the cap head closes fully.

1.8.7. Repeat steps 1.8.1. through 1.8.6. for the other actuator spring.

1.9. Placing the leg pads in hip frame.

Plan 1.9. If you are a novice user skip step 1.9.5. only. If you are a familiar/expert user skip step 1.9.4. only.

1.9.1. Identify the right leg pad. The right leg pad forms an ‘L’ when seen from the front.

1.9.2. Put the leg pad fixator in the slots of the leg pad.

1.9.3. Put the leg pad fixator in the slot of the right smart joint.

1.9.4. Move the leg pad to the upper or lower setting in the smart joint.

1.9.5. Put two screws in the two holes of the smart joint.

1.9.6. Use a 3 mm hex key to tighten until the screw heads contact the smart joint.

1.9.7. Do steps 1.9.1. through 1.9.6. again, for the left leg pad.

1.10. Installing the leg straps.

1.10.1. Make sure that the buckle on the leg strap is facing outward.

1.10. 2. Put the end of the strap without the buckle through the slot in the leg shell.

1.10.3. Repeat 1.10.1. and 1.10. 2. for other leg shell.

2. Don the Laevo Flex

Plan 2. Do 2.1. if necessary. Then do only 2.2 and 2.3. if all is well adjusted (not first-time use). Otherwise, do 2.1. if necessary. Then, do 2.2. If necessary, after 2.2.3. disconnect torso structures and skip to 2.4. Then, return to 2.2.3., finish 2.3. and continue with 2.5., 2.6., 2.7., and 2.8., if any of these need completion, if not, skip unnecessary step. Then, do 2.9.

2.1. User preparation before donning the Laevo Flex

Plan 2.1. Only complete steps that are applicable.

2.1.1. Empty your pockets.

2.1.2. Remove uncomfortable accessories and clothing, for example, necklaces or scarves.

2.1.3. Long hair must be tied back.

2.1.4. Make sure that the screw caps that lock the actuator springs in the smart joints are tight.

2.2. Put on Laevo Flex

2.2.1. Put on the vest.

2.2.2. Keep the vest open.

2.2.3. Place the hip frame around your hips with the smart joints positioned on your hip joints.

2.2.4. Close the front belt.

2.2.5. Put the leg shells on your thighs.

2.2.6. Close the zipper of the vest.

2.3. Adjust the front vest

2.3.1. Set the straps to a long, loose fit.

2.3.2. Put on the vest.

2.3.3. Close the zipper.

2.3.4. Take a semi-deep breath.

2.3.5. Tighten the lower front vest straps to a comfortable setting.

2.3.6. Tighten the upper front vest straps to a comfortable setting.

2.3.7. Wear the vest at a comfortable height.

2.3.7.1. Check that the vest covers your sternum.

2.3.7.2. Check that the vest does not press your stomach.

2.3.7.3. Check that the vest allows you to reach forward comfortably.

2.3.7.4. Check that the vest does not cut into your neck.

2.3.8. Ensure the following steps are met:

2.3.8.1. The end of the strap overlaps the Velcro of the vest.

2.3.8.2. You can breathe comfortably.

2.3.8.3. The left and right vest straps are the same length.

2.3.9. Check that the Velcro is attached correctly.

2.4. Align the hip frame and smart joints.

2.4.1 Feel where your hip joint (the bone part that can be felt on the outside) is located by turning your heel on the ground.

2.4.2. Loosen all the belts of the hip frame.

2.4.3. Put on the hip frame and pull outward to widen the frame.

2.4.4. Put the smart joints on hip joints. Put the round indication on the hip frame over hip joint (the bone part that can be felt on the outside).

2.4.5. Tighten the upper back belt until it presses against your back.

2.4.6. Tighten the front belt.

2.4.7. Get into a semi-squat position / bend your knees and slightly tighten the bottom belt.

2.4.8. Stand straight.

2.4.9. Raise one leg and examine the smart joints to make sure that they are aligned with your hip joints.

2.4.10. Move the sleeve over the front buckle.

2.5. Adjust the length of the torso structure

Plan 2.5. Do 2.5.1. through 2.5.4. in order. Then, if the swivel arm points down and pulls the vest down do 2.5.4 and 2.5.5. On the other hand, if the swivel arm points up and pushes the vest up do 2.5.6. and 2.5.7. instead. Steps 2.5.4. through 2.5.7. can only be completed if someone is helping the main user or if the Laevo Flex is not worn.

2.5.1. Stand straight and relaxed, with the FLEX on.

2.5.2. From the side, check the angle of the back connector swivel. The swivel arm (for back connector) must be horizontal or point slightly downward.

2.5.3. Feel if vest is being pushed up or pulled down.

2.5.4. Remove the torso structure from the smart joint by pushing the button.

2.5.4. Adjust the torso structure lengths to a larger setting.

2.5.5. Make sure that the height setting is the same on the left and right sides.

2.5.6. Adjust the torso structure lengths to a shorter setting.

2.5.7. Make sure that the height setting is the same on the left and right sides.

2.6. Fit leg pads

Plan 2.6. Do 2.6.1. and 2.6.2. concurrently. Then, if the leg shells are far above the knees and cause discomfort do only 2.6.3 and skip 2.6.4. However, if the leg shells are on the knees and you have knee problems or experience discomfort do only 2.6.4. and skip 2.6.3. Then, if the leg shells are not centered side to side on the thighs, and the leg shells move sideways during use and cause discomfort do 2.6.5.

2.6.1. Make sure that the leg shells push on the legs while slightly stooped.

2.6.2. Make sure the lower rim of the leg shells is above the kneecaps. The recommended distance is 2 to 5 cm.

2.6.3. Adjust the length of the leg pads to a larger setting or select a larger leg pad size.

2.6.4. Adjust the length of the leg pads to a smaller setting or select a smaller leg pad size

2.6.5. Attach leg straps.

2.7. Fit leg straps

2.7.1. Put the leg shell in a comfortable position on your leg.

2.7.2. Use the clip on the leg strap to connect the leg strap to the leg shell.

2.7.3. Pull on the end of the leg strap to tighten the leg strap.

2.8. Set angle of the smart joint

Plan 2.8. Do 2.8.1 through 2.8.5. for one leg pad, then repeat these steps for the other leg pad. Then, do 2.8.6. and 2.8.7. Then, if true that the leg shells lift off of your thighs, do 2.8.1 through 2.8.7. then do 2.8.8. On the other hand, if true that the leg shells press uncomfortably on thighs do 2.8.1 through 2.8.7. then do 2.8.9.

2.8.1. Stand in a natural, straight position.

2.8.2. Turn the leg pads off your thighs.

2.8.3. Squeeze the smart joint to unlock the angle setting.

2.8.4. Turn the leg pad, while you squeeze the smart joint.

2.8.5. Release the smart joint. An audible click means the angle setting is locked.

2.8.6. Make sure that the setting for the angle of the smart joint is the same on both sides.

2.8.7. Put the leg pads back on your thighs.

2.8.8. Set the angle of the smart joint to a higher setting.

2.8.9. Set the angle of the smart joint to a lower setting.

2.9. Check the overall fit of the Laevo Flex.

2.9.1. Maintain a straight but relaxed posture when standing.

2.9.1.1. Ensure that the swivel arm is horizontal or points slightly downward.

2.9.1.2. Ensure that the left and right sides are approximately symmetrical.

2.9.1.3. Ensure that the back connector swivel is horizontal.

2.9.1.4. Ensure that the vest straps are adjusted symmetrically.

2.9.1.5. Ensure that the remaining length of the belt is rolled up.

2.9.1.6. Ensure that the buckle of the front belt is covered by the front belt sleeve.

2.9.1.7. Make sure you feel the smart joints on your hip joints.

2.9.1.8. Make sure you feel the leg shells press slightly on your thighs or lift off your thighs slightly.

2.9.1.9. Make sure you feel the hip frame feels secure and that the hip frame does not press uncomfortably.

2.9.1.10. Make sure you feel all belts of the hip frame are tight.

2.9.1.11. Make sure you feel the vest feels secure. The vest does not press the chest uncomfortably.

2.9.1.12. Make sure that your arms can move freely.

3. Doff Laevo Flex

Plan 3. Do 3.1., 3.2.3.3., 3.4, 3.5 and 3.6. in order. If leg straps are not used, skip 3.2.

3.1. Open the zipper of the vest. (Unzip)

3.2. Remove the leg straps from leg shells.

3.3. Remove the leg shells from your thighs.

3.4. Open the front belt. (Unbuckle)

3.5. Remove the hip frame from around your hips.

3.6. Take off the vest.

4. Disassemble Laevo Flex

Plan 4. Do 4.1. Then, either first do 4.2., 4.3., 4.4., and 4.5. or first do 4.6., 4.7. Only do 4.8. if leg straps were used. Also, the user can decide to skip 4.3., 4.5., and 4.6.

4.1. Detach torso structures from hip frame.

Plan 4.1. Do 4.1.1. and 4.1.2. for one side. Then, repeat 4.1.1. and 4.1.2. for the other side.

4.1.1. Push down and hold on the button in the smart joint to detach torso structure from hip frame.

4.1.2. Pull on torso structure slightly to separate the two parts.

4.2. Disconnect torso structures from back connector.

Plan 4.2. Do 4.2.1. and 4.2.2. for one side. Then, repeat 4.2.1. and 4.2.2. for the other side.

4.2.1. Push down and hold on the button in the back connector located on top of the torso structure.

4.2.2. Pull the torso structure down to disconnect.

4.3. Detach back connector from lower bar.

4.3.1. Push down on circle silver button on the side of the body of the back connector.

4.3.2. Tilt the back connector from right to left while lifting it up.

4.4. Remove front vest.

4.4.1. Close the zipper of the front vest.

4.4.2. Open the Velcro of the front vest straps (4).

4.4.3. Remove the front vest straps from the slots of the vest frame (4) by pulling it out of lower bar.

4.5. Remove vest frame padding.

4.5.1. Open the Velcro of the bottom of the vest padding.

4.5.2. Open the Velcro of the top of the vest padding.

4.5.3. Remove the vest padding from the vest frame.

4.6. Take out actuator springs.

Plan 4.6. Do 4.6.1. through 4.6.5. for one side. Then, repeat steps 4.6.1. through 4.6.5. for other side.

4.6.1. Use an 8 mm hex key to remove the cap from the smart joint.

4.6.2. Remove the actuator spring from the smart joint.

4.6.3. Put the cap in the hole at the top of the smart joint.

4.6.4. Use the supplied 8 mm hex key to tighten until the cap head contacts the smart joint.

4.6.5. Check that the cap head closes fully.

4.7. Remove leg pads.

Plan 4.7. Do 4.7.1. through 4.7.5. for one side. Then, repeat steps 4.7.1. through 4.7.5. for other side.

4.7.1. Use a 3 mm hex key to loosen both screws.

4.7.2. Remove screws.

4.7.3. Remove the leg pad fixator from the slot of the smart joint.

4.7.4. Remove the leg pad fixator from the slot of the leg pad.

4.7.5. Screw on the screws to fixator.

4.8. Remove leg straps.

Plan 4.8. Do 4.8.1. for both sides.

4.8.1. Disconnect clip from leg shell.

SKELEX

0. Assemble, don, doff, and disassemble Skelex

Plan 0. Do 1, 2, 3, 4 in order.

1. Assemble the Skelex

1.1. Measure user and complete adjustments in skelex.

1.1.1 Measure waist from your left side to your right side in cm and write down the measurement.

1.1.2. Adjust the size of the belt, push in, or pull out the belt depending on the corresponding waist size.

1.1.2.1. Stop adjusting once the corresponding size is next to the indicator in the inner center of the belt.

1.1.3. Measure in a straight line the vertical distance from the hip bone to the top of your shoulder in cm and write down the measurement. 43

1.1.4. Adjust the height of the frames.

1.1.4.1. Locate the spring height adjustment at the bottom of the flex frame.

1.1.4.2. Hold the lower frame in one hand and pinch both adjustment knobs with the other hand.

1.1.4.3. Slide the lower frame up or down until the measurement taken in 1.1.3. is visible in the window.

1.1.4.4. Release the pinch on the adjustment knobs and repeat for the other flex frame.

1.1.5. Adjust the arm length.

1.1.5.1. Identify the letter (S, M, L) from the previous step to set the arm length correctly.

1.1.5.2. Push the blue knobs towards each other to release the mechanism and slide the arm interface until the corresponding length (S, M, or L) is visible in the window.

1.1.5.3. Release the adjustment knobs and repeat for the other arm.

1.1.6. Measure body weight on scale.

1.1.7. Adjust support force settings.

1.1.7.1. Select the corresponding force adjustment setting according to the body weight.

1.1.7.1.1. Select adjustment setting 1 If body weight is between 50 to 64 kg / 110 to 141 lb.

1.1.7.1.2. Select adjustment setting 2 If body weight is between 65 to 79kg / 142 to 174 lb.

1.1.7.1.3. Select adjustment setting 3 If body weight is between 80 to 95 kg / 175 to 207 lb.

1.1.7.1.4. Select adjustment setting 4 If body weight is above 95 kg / 208 lb.

1.1.7.2. Open the blue lever by putting it down.

1.1.7.3. Rotate the lever until the force indicator is at the desired position.

1.1.7.4. Place the blue lever back in the housing.

1.1.7.5. Repeat for the other arm.

1.1.8. Measure the circumference of the upper arm.

1.1.8.1. Flex arm to make a 90 degree angle, having hand pointing upwards.

1.1.8.2. Keep the upper arm horizontal and take the measurement halfway the upper arm or on the largest part of the bicep muscle.

1.1.9. Select arm cup size.

1.1.9.1. Select a small arm cup if your arm circumference is between 20 and 28 cm.

1.1.9.2. Select a medium arm cup if your arm circumference is between 27 and 35 cm.

1.1.9.3. Select a large arm cup if your arm circumference is between 34 and 42 cm.

1.1.9.4. Select the larger size for arm cup if the are two possible sizes.

1.1.9.5. Select the arm cup size closest to the arm measurement if the corresponding size is not stated above.

1.2. Loop the lower portion of the left flex frame all the way through the left loose strap in the back of the harness.

1.3. Insert the left flex frame into the belt connector and rotate it 180° until a click sound is heard.

1.4. Attach the harness connector from the left flex frame to the Minax (black plastic tube on harness).

1.4.1. Pull down the Minax lock.

1.4.2. Insert the harness connector into the Minax.

1.4.3. Push up the Minax lock to secure the connection between harness and flex frame.

1.5. Place an arm cup into the left flex frame by pushing down the blue buttons and simultaneously inserting the arm cup into the socket by twisting until a click sound is heard.

1.6 Check if the arm cup is placed correctly by pulling on the arm cup to make sure it is locked in place.

1.7. Repeat steps 1.2. through 1.6. for the right side.

2. Don the skelex

2.1. Open the Velcro belt.

2.2. Open the chest strap buckle.

2.3. Loosen the shoulder straps.

2.4. Put the exoskeleton on like a backpack.

2.5. Put it loosely on shoulders.

2.6. Strap the belt with some tension having the elastic part on the hip.

2.6.1. Make sure the belt is symmetrically on your left and right hip.

2.7. Engage and lock the chest strap.

2.7.1. Secure the chest strap at a comfortable height.

2.7.2. Tighten the chest strap so the shoulder straps are away from the armpit.

2.8. Adjust the shoulder straps.

2.8.1. Pull the shoulder straps and tighten them for a comfortable fit.

2.9. Secure back shoulder strap and re-route the end of the shoulder strap webbing through the buckle.

2.9.1. Widen the back shoulder strap, if you experience pressure or discomfort on the shoulders due to contact.

2.9.2. Tighten the back shoulder straps if the flex frames are away from your shoulders.

2.10. Check with the arm down the distance between the spring and the shoulder, to ensure it is 2 fingers thick.

2.11. Engage the arm cups by pulling the GripLabel (yellow tab with a ‘4’) at the end of the arm cup strap down.

2.12. Place your upper arm on the arm cup.

2.13. Wrap the strap around your upper arm.

2.14. Lock the grip label on the hook of the arm cup.

2.15. Make sure the grip label is securely locked in place

2.15.1. Make sure there is enough tension in the arm straps to avoid unlocking the grip label.

2.15.2. Avoid too much tension in the straps to prevent pinching of the upper arm by the arm straps.

2.15.3. Make sure that the arm cup straps is evenly spread on the upper arm and create a “V” shape.

2.16. Repeat 2.11. through 2.15. for the other arm cup.

3. Doff the skelex

Plan 3. If shoulder straps are not tight skip 3.4. Else, do all in order.

3.1. Disengage one arm cup from arm carefully by unlocking the grip label from the arm cup hook.

3.1.1. Remove arm from arm cup in controlled moves as it will bounce back up.

3.2. Disengage the other arm cup from arm carefully by unlocking the grip label from the arm cup hook.

3.1.2. Remove arm from arm cup in controlled moves as it will bounce back up.

3.3. Unbuckle chest strap.

3.4. Loosen shoulder straps.

3.5. Unstrap belt.

3.6. Remove the exoskeleton like a backpack and place it the working surface.

4. Disassemble the Skelex

4.1. Pull down Minax (black tubes in harness) lock at the underside of the top of the spring.

4.2. Detach the harness connector from Minax.

4.3. Rotate the flex frame 180° and pull out of the belt connector to remove the frame.

4.4. Remove the harness loop from the tube part of the height adjustment.

4.5. Press down on the blue buttons next to the arm cup with some force and simultaneously wiggle the arm cup out by pulling on it.

4.6. Repeat 4.1. through 4.5. for other side.
